# Supplementary material for: Involvement of condensin in cellular senescence through gene regulation and compartmental reorganization
Source: Nat Commun. 2019 Dec 12;10:5688. doi: 10.1038/s41467-019-13604-5 (PMC6908677; doi:10.1038/s41467-019-13604-5)
Supplement: Supplementary file 1 — Supplementary Information [file 41467_2019_13604_MOESM1_ESM.pdf]

SUPPLEMENTARY INFORMATION

Involvement of Condensin in Cellular Senescence through Gene Regulation and Compartmental Reorganization

Osamu Iwasaki, Hideki Tanizawa, Kyoung-Dong Kim, Andrew Kossenkov, Timothy Nacarelli, Sanki Tashiro, Sonali Majumdar, Louise C. Showe, Rugang Zhang, and Ken-ichi Noma*

*Correspondence should be addressed to:

E-mail: noma@uoregon.edu

Contents

Supplementary Figures 1-12
Supplementary Tables 1-3
Supplementary Notes
Supplementary Methods
Supplementary References

Supplementary Figure

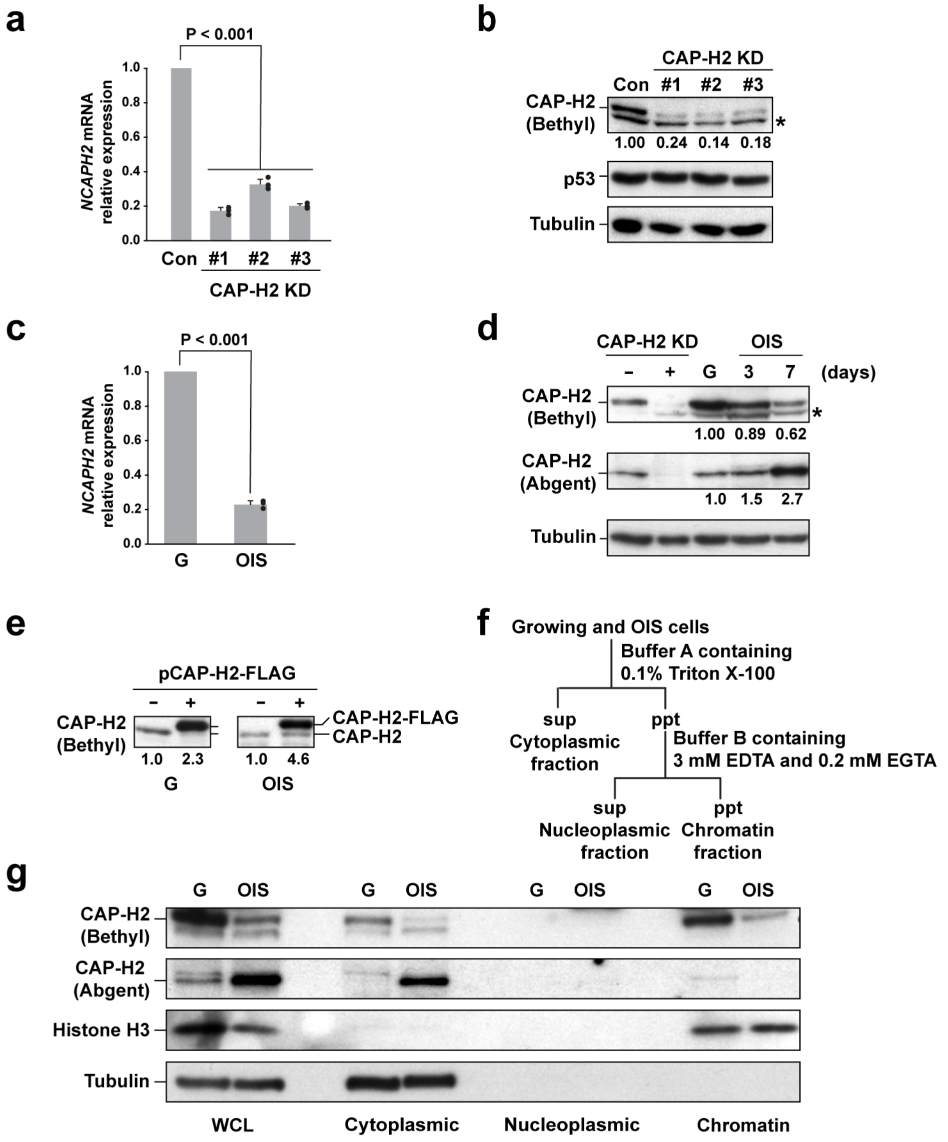

Supplementary Figure 1. Detection of the condensin II subunit, CAP-H2, in OIS and growing cells

(**a**) RT-qPCR analysis to examine the efficiency of CAP-H2 knockdown (KD). IMR90 cells were infected with retrovirus encoding H-RasV12 and cultured for 7 days to establish OIS (**Methods**). OIS cells were further infected with lentivirus encoding one of the three shRNA constructs (#1, #2, and #3) against *NCAPH2* or carrying an empty vector (control) and harvested 3 days after the lentivirus infection. *P* values were calculated by two-sided Student's *t* test, using biologically independent samples (n = 3, error bars represent the SD).

(**b**) Whole cell lysates of OIS cells with and without CAP-H2 KD were subjected to western blot analysis. CAP-H2 antibody (Bethyl Laboratories, A302-275A) was used for immunoblotting.

Tubulin serves as a loading control. Asterisk indicates non-specific band. Relative expression of CAP-H2 proteins after CAP-H2 KD (#1, #2, and #3) compared to expression without KD (control) was estimated.

(**c**) RT-qPCR to investigate *NCAPH2* mRNA in growing (G) and OIS cells. *P* values were calculated by two-sided Student's *t* test, using biologically independent samples (n = 3, error bars represent the SD).

(**d**) Whole cell lysates were prepared from growing cells with and without CAP-H2 KD (left two lanes) and also from growing and OIS cells (right three lanes). Cells were harvested on the indicated days after infection of H-RasV12 retrovirus. The two CAP-H2 antibodies (Bethyl Laboratories, A302-275A; Abgent, AP1973A) were used for immunoblotting.

(**e**) Detection of endogenous CAP-H2 and exogenous CAP-H2-FLAG proteins. Lysates of growing and OIS cells with (+) and without (-) infection of retrovirus encoding CAP-H2-FLAG were subjected to western blotting. Expression of exogenous CAP-H2-FLAG proteins was compared to that of endogenous CAP-H2 proteins without the retroviral infection.

(**f**) Growing and OIS cells were subjected to chromatin fractionation[1]. Buffer A contains 10 mM Hepes-KOH (pH 8.0), 10 mM KCl, 1.5 mM $MgCl_2$, 0.34 M Sucrose, 10% Glycerol, EDTA-free Protease Inhibitor Cocktail (Roche), 1 mM DTT, 0.1 mM PMSF, and 0.1% Triton X-100; Buffer B contains 3 mM EDTA, 0.2 mM EGTA, EDTA-free Protease Inhibitor Cocktail, 1 mM DTT, and 0.1 mM PMSF.

(**g**) Whole cell lysates (WCL), cytoplasmic fraction, nuclear soluble fraction (nucleoplasmic), and chromatin-bound fraction were prepared from growing and OIS cells as explained in panel **f** and subjected to western blot analysis.

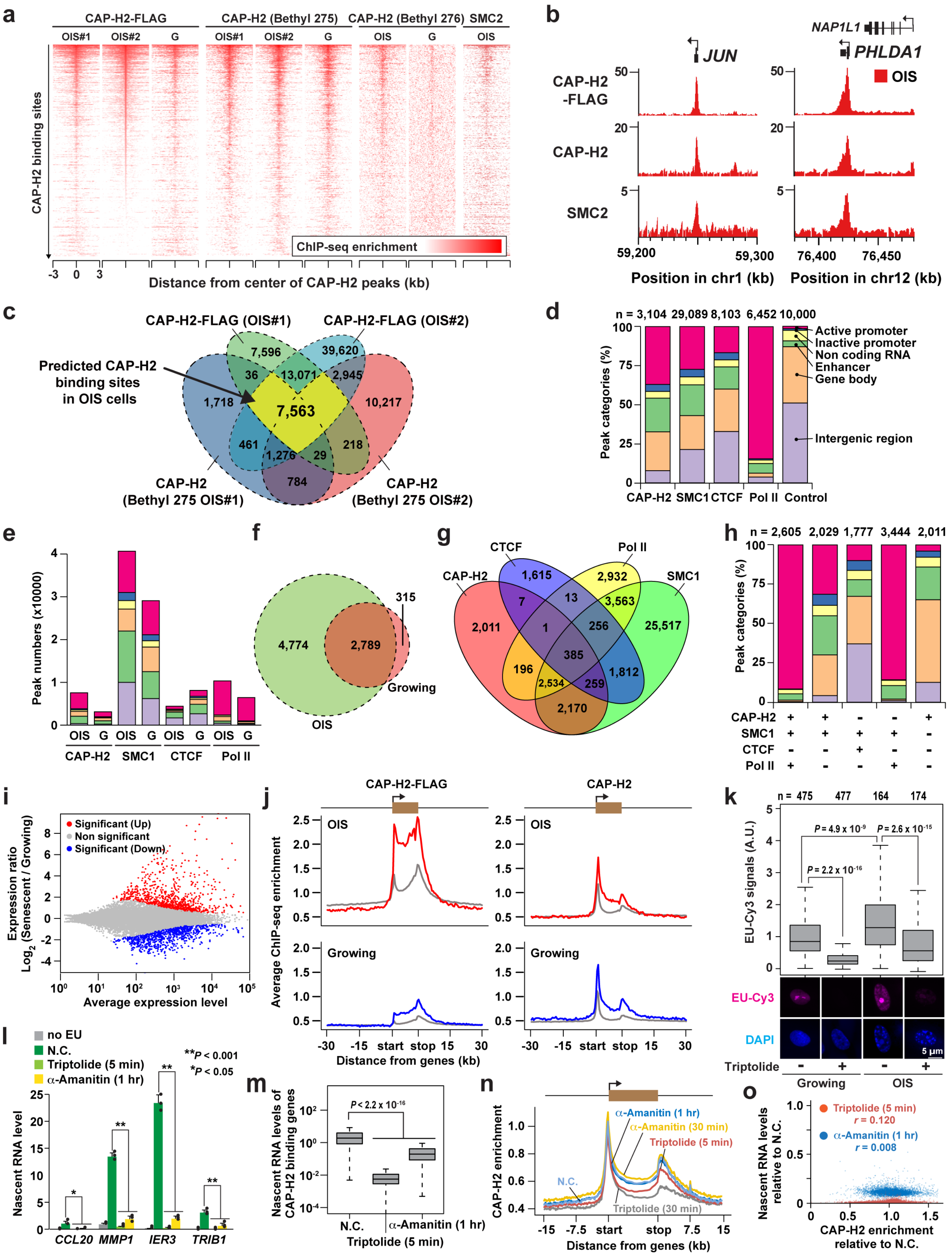
a
CAP-H2-FLAG
CAP-H2 (Bethyl 275)
CAP-H2 (Bethyl 276)
SMC2
OIS#1
OIS#2
G
OIS
CAP-H2 binding sites
ChIP-seq enrichment
-3
0
3
Distance from center of CAP-H2 peaks (kb)
b
NAP1L1
JUN
PHLDA1
OIS
CAP-H2 -FLAG
CAP-H2
SMC2
50
20
5
59,200
59,300
76,400
76,450
Position in chr1 (kb)
Position in chr12 (kb)
c
CAP-H2-FLAG (OIS#1)
CAP-H2-FLAG (OIS#2)
Predicted CAP-H2 binding sites in OIS cells
7,596
39,620
36
13,071
2,945
1,718
7,563
10,217
461
218
1,276
29
784
CAP-H2 (Bethyl 275 OIS#1)
CAP-H2 (Bethyl 275 OIS#2)
d
n = 3,104 29,089 8,103 6,452 10,000
Peak categories (%)
Active promoter
Inactive promoter
Non coding RNA
Enhancer
Gene body
Intergenic region
CAP-H2
SMC1
CTCF
Pol II
Control
e
Peak numbers (x10000)
OIS
G
CAP-H2
SMC1
CTCF
Pol II
f
315
4,774
2,789
Growing
OIS
g
CTCF
Pol II
CAP-H2
SMC1
1,615
2,932
7
13
3,563
2,011
1
256
25,517
385
196
1,812
2,534
259
2,170
h
n = 2,605 2,029 1,777 3,444 2,011
Peak categories (%)
CAP-H2
SMC1
CTCF
Pol II
i
Significant (Up)
Non significant
Significant (Down)
Expression ratio Log2 (Senescent / Growing)
Average expression level
j
CAP-H2-FLAG
CAP-H2
OIS
Growing
Average ChIP-seq enrichment
-30
-15
start
stop
15
30
Distance from genes (kb)
k
n = 475 477 164 174
P = 4.9 x 10-9
P = 2.6 x 10-15
P = 2.2 x 10-16
EU-Cy3 signals (A.U.)
EU-Cy3
DAPI
5 µm
Triptolide
Growing
OIS
l
no EU
N.C.
Triptolide (5 min)
α-Amanitin (1 hr)
**P < 0.001
*P < 0.05
Nascent RNA level
CCL20
MMP1
IER3
TRIB1
m
P < 2.2 x 10-16
Nascent RNA levels of CAP-H2 binding genes
N.C.
α-Amanitin (1 hr)
Triptolide (5 min)
n
α-Amanitin (1 hr)
α-Amanitin (30 min)
Triptolide (5 min)
N.C.
Triptolide (30 min)
CAP-H2 enrichment
-15
-7.5
start
stop
7.5
15
Distance from genes (kb)
o
Triptolide (5 min)
r = 0.120
α-Amanitin (1 hr)
r = 0.008
Nascent RNA levels relative to N.C.
CAP-H2 enrichment relative to N.C.

Supplementary Figure 2. Condensin binding sites in OIS and growing cells

(**a**) CAP-H2 binding peaks in OIS and growing (G) cells determined using the CAP-H2 Bethyl 275 and 276 antibodies (Bethyl Laboratories, A302-275A and A302-276A) and anti-FLAG M2 monoclonal antibody (**Methods**). OIS #1 and OIS #2 indicate biological replicas. CAP-H2-FLAG (OIS #1) binding peaks were ranked by CAP-H2-FLAG enrichment in OIS cells, and CAP-H2-FLAG binding loci with closest enrichment scores were categorized into 400 groups. Average ChIP-seq enrichment for each group was plotted around ±3 kb regions from the midpoint of CAP-H2-FLAG-enriched peaks. Binding peaks of an SMC2 condensin subunit in OIS cells were also determined by ChIP-seq and subjected to the same analysis.

(**b**) Co-localization of CAP-H2 and SMC2 in OIS cells.

(**c**) CAP-H2 binding sites in OIS cells (yellow sections in the Venn diagram) were predicted from the indicated CAP-H2 ChIP-seq data (**Supplementary Notes**).

(**d**) Distributions of CAP-H2, SMC1, CTCF, and Pol II binding sites at the indicated genetic elements, in growing cells. Numbers of total binding sites for the indicated proteins are shown at top. For the control, 10,000 loci were randomly selected from the entire genome and classified into the same categories.

(**e**) Comparison of CAP-H2, SMC1, CTCF, and Pol II binding sites between OIS and growing cells. Colors represent the genetic elements annotated in panel **d**.

(**f**) Overlap of CAP-H2 binding peaks in OIS and growing cells.

(**g**) Overlap among binding sites of the indicated factors in OIS cells.

(**h**) Co-occupancy of genomic regions by the same factors as in panel **g**. Colors represent the genetic elements annotated in panel **d**.

(**i**) Changes in gene expression in OIS cells compared to growing cells, as determined by RNA-seq data. Expression ratios between OIS and growing cells (Y axis) are plotted against average expression levels for respective genes (X axis). Red and blue dots indicate significantly up- (n = 842) and down-regulated (n = 865) genes, respectively (**Methods**).

(**j**) Average CAP-H2-FLAG and CAP-H2 binding patterns at the significantly up- (top, red, n = 808) and down- (bottom, blue, n = 842) regulated genes in OIS cells compared to growing cells; only genes > 2 kb were selected for analysis. Grey lines show average binding patterns at genes without significantly altered expression (n = 15,893 among 19,591 genes).

(**k**) Nascent transcripts visualized in OIS and growing cells. Cells were treated by an RNA polymerase II inhibitor, Triptolide (1 μM), for 1 hour and subsequently incubated with 5-ethynyl

uridine (EU) for additional 1 hour. EU was incorporated into nascent RNA and reacted with Cy3-azide via Click chemistry. This analysis was performed as detailed in **Supplementary Methods**, and distributions of nuclear nascent RNA signals are shown as boxplots (central bar represents the median with boxes indicating the upper and lower quartiles, and whiskers extend to the data points, which are no more than 1.5x the interquartile range from the box; two-sided Mann–Whitney *U* test). A.U., arbitrary unit.

(**l**) Effect of RNA polymerase inhibitor treatment (Triptolide and α-Amanitin) on nascent RNA levels determined by RT-qPCR. OIS cells were treated by the inhibitors and incubated with EU. EU-incorporated nascent transcripts were reacted with biotin-azide via Click chemistry, purified using streptavidin beads, and subjected to RT-qPCR (**Supplementary Methods**). *P* values were calculated by two-sided Student's *t* test, using biologically independent samples (n = 3, error bars represent the SD).

(**m**) Effect of RNA polymerase inhibitor treatment on nascent RNA levels determined by RNA-seq. OIS cells were treated with the RNA polymerase inhibitors, and nascent RNA prepared as in panel **l** were subjected to RNA-seq. Nascent RNA levels at CAP-H2 binding genes (n = 7,622) were determined as described in **Supplementary Methods** and plotted as boxplots (central bar represents the median with boxes indicating the upper and lower quartiles, and whiskers extend to the data points, which are no more than 1.5x the interquartile range from the box; two-sided Mann–Whitney *U* test).

(**n**) Effect of RNA polymerase inhibitor treatment on CAP-H2 binding. Inhibitor-treated OIS cells were subjected to CAP-H2 ChIP-seq analysis. Average CAP-H2 binding levels at CAP-H2 binding genes (n = 7,622) were calculated.

(**o**) Relationship between nascent RNA levels (Y axis) and CAP-H2 ChIP-seq enrichment (X axis). Nascent RNA levels and CAP-H2 enrichment at CAP-H2 binding genes in OIS cells treated by Triptolide (5 minutes) and α-Amanitin (1 hour) were normalized by those without the inhibitor treatment. Pearson's correlation coefficient (*r*) between relative nascent RNA levels and CAP-H2 ChIP-seq enrichment was indicated.

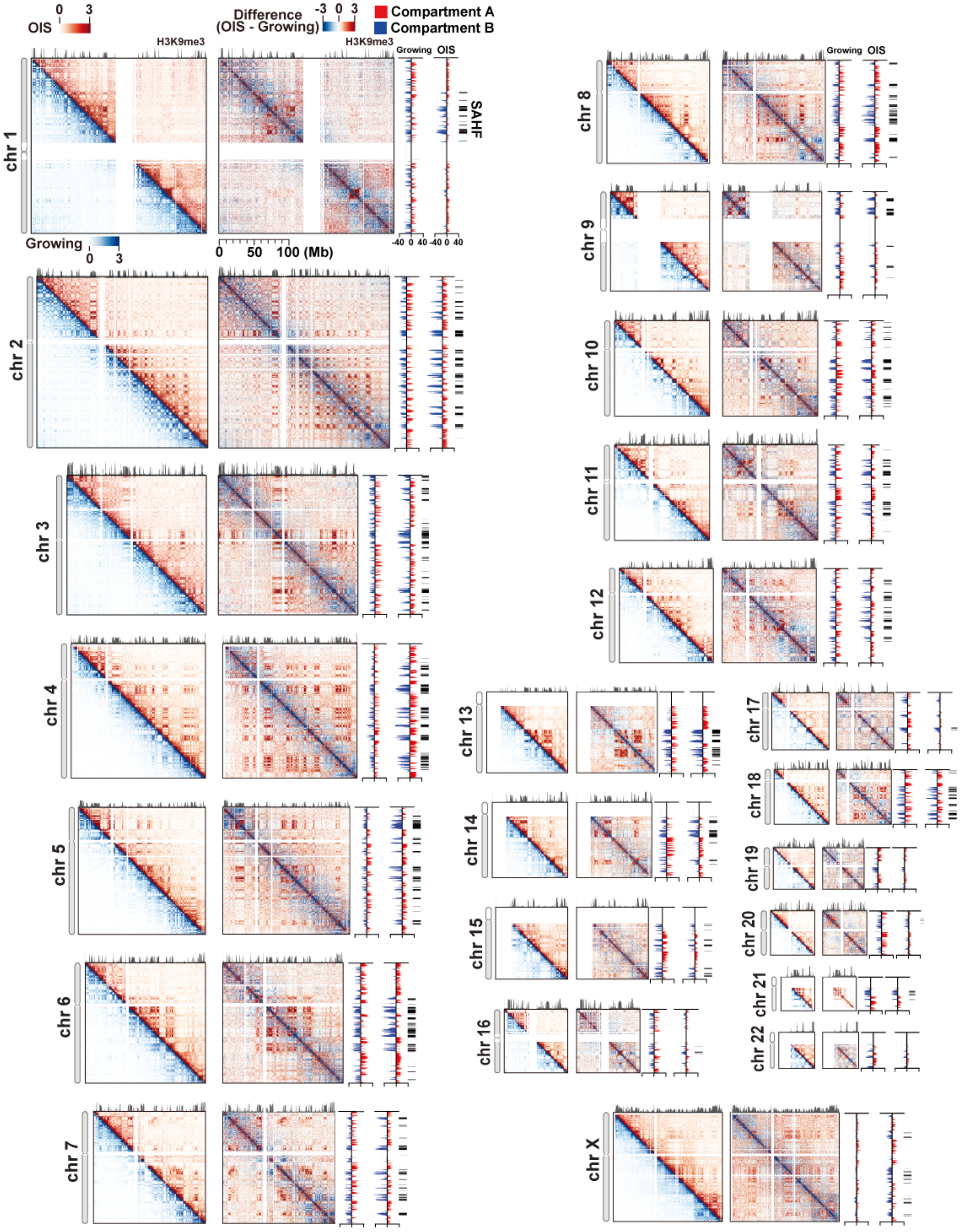
OIS
0
3
Difference
(OIS - Growing)
-3
0
3
Compartment A
Compartment B
H3K9me3
H3K9me3
Growing
OIS
SAHF
Growing
0
3
-40 0 40 -40 0 40
0
50
100 (Mb)
chr 1
chr 2
chr 3
chr 4
chr 5
chr 6
chr 7
chr 8
chr 9
chr 10
chr 11
chr 12
chr 13
chr 14
chr 15
chr 16
chr 17
chr 18
chr 19
chr 20
chr 21
chr 22
chr X

Supplementary Figure 3. Genome-wide contact maps at 200 kb resolution in OIS (top right, red) and growing (bottom left, blue) cells

Contact maps were generated as described in **Methods**. Histone H3K9me3 ChIP-seq data (GEO accession#, GSE38448[https://www.ncbi.nlm.nih.gov/geo/query/acc.cgi?acc=GSE38448]) in growing cells are shown at top. Note that global H3K9me3 patterns were similar between OIS and growing cells[2]. Difference of contact probabilities between OIS and growing cells are also shown as described in **Fig. 2b**. PCA scores in growing and OIS cells are shown to the right as described in **Fig. 2c**.

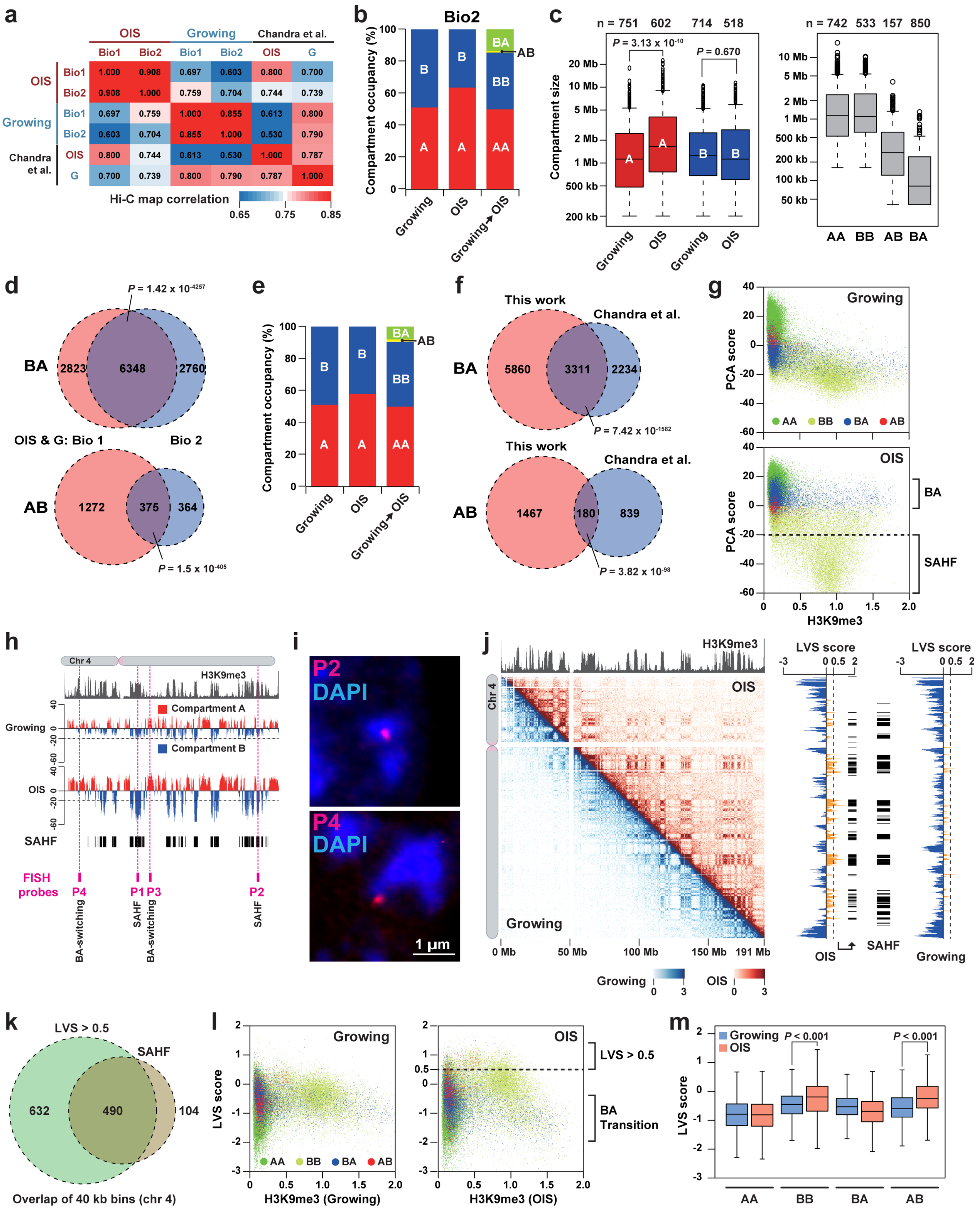

Supplementary Figure 4. SAHF formation and compartmental reorganization upon OIS

(**a**) Reproducibility of in situ Hi-C data. Correlation between the indicated contact maps was evaluated using the HiCRep program[3]. Contact maps at 500 kb resolution were compared, where genomic combinations within 100 Mb were included in the estimation. Bio indicates biological replicas. Contact maps from OIS and growing WI-38 cells[4] were generated using the same procedure as for the in situ Hi-C data.

(**b**) Occupancy of A and B compartments in growing and OIS cells (for replicate #2). The human genome was divided into 40 kb bins, which were assigned either to A or B compartments. Right column shows the composition of the compartmental categories (AA, BB, AB, and BA) upon switching from growing to OIS. For instance, BA indicates B compartments in growing cells that switched to A compartments in OIS cells.

(**c**) Left: Size distributions of A and B compartments for growing and OIS cells shown as boxplots (central bar represents the median with boxes indicating the upper and lower quartiles, and whiskers extend to the data points, which are no more than 1.5x the interquartile range from the box, outliers shown as circles, two-sided Mann–Whitney *U* test; same data as panel **b**). Numbers of A and B compartments are shown at top. Right: Size distributions and numbers of genomic regions that belong to the respective compartmental categories.

(**d**) Overlap between biological replicates (Bio 1 and Bio2) of genomic sections (40 kb bins) that show BA or AB transitions (hypergeometric distribution test). To estimate BA or AB transitions, A/B compartments in the OIS Bio1 and Bio2 data were compared with those in the growing Bio1 and Bio2 data.

(**e**) Occupancy of A and B compartments in growing and OIS WI-38 cells[4]. Right column shows the composition of the compartmental categories (AA, BB, AB, and BA) upon switching from growing to OIS.

(**f**) Top: Overlap of BA transitions upon OIS in IMR90 cells (OIS Bio1 and G Bio1) and in WI-38 cells[4]. Bottom: Overlap of AB transitions upon OIS in IMR90 and WI-38 cells. The analysis was performed as described in panel **d** (hypergeometric distribution test).

(**g**) Correlation between PCA score, H3K9me3 enrichment and chromatin compartment for all 40 kb genomic regions across the genome, in growing and OIS cells (for Bio2 data). Data was analyzed as in **Fig. 2f** (Bio1 data). Dot colors represent genomic regions belonging to the indicated compartmental categories. SAHF regions are defined as PCA scores < -20.

(**h**) Probe positions employed for FISH experiments. The SAHF-forming heterochromatic (P1 and P2 probes) and BA-switching loci (P3 and P4) were visualized in OIS cells.

(**i**) Examples of FISH images for the P2 and P4 probes. SAHF were visualized by DAPI staining.

(**j**) Left: Contact maps for chromosome 4 in OIS (top right) and growing cells (bottom left) at 200 kb resolution (duplicated from **Fig. 2a**). Right: LVS scores for each 40 kb region, calculated based on contact probabilities in OIS and growing cells; LVS scores reflect the frequency of long-range contacts (>2 Mb) (**Supplementary Methods**). LVS scores > 0.5 (orange peaks) are annotated as black bars; locations of SAHF regions (duplicated from **Fig. 2c**) are shown to the right for comparison.
(**k**) Overlap between PCA-defined SAHF regions and genomic sections with LVS scores > 0.5. The human genome was divided into 40 kb bins, and genomic sections having LVS scores > 0.5 and PCA scores < -20 were compared.
(**l**) Correlation between LVS score, H3K9me3 enrichment and chromatin compartment, for every 40 kb bin across the human genome, for growing and OIS cells. Regions with LVS scores > 0.5 are indicated.
(**m**) Distributions of LVS scores (from panel **l**) grouped by compartmental category shown as boxplots (central bar represents the median with boxes indicating the upper and lower quartiles, and whiskers extend to the data points, which are no more than 1.5x the interquartile range from the box; two-sided Mann–Whitney *U* test).

a

	AA	BB	AB	BA
CAP-H2	0.22	-0.36	-0.35	0.00
SMC1	0.26	-0.45	-0.40	0.06
LMNB1	-0.35	0.26	-0.06	0.40
H3K9me3	-0.46	0.51	-0.43	-0.17
H3K9me2	-0.02	-0.05	-0.07	0.22
H3K27me3	0.23	-0.54	-0.16	0.35
H3K4me3	0.67	-1.52	-0.82	-0.65
H3K36me3	0.64	-1.50	-0.15	-0.61

log_2 (OIS / Con) -1 0 1

b

	AA	BB	AB	BA
CAP-H2	-0.07	0.03	-0.32	0.25
SMC1	0.03	-0.06	-0.16	0.07
LMNB1	-0.06	0.07	0.14	0.04
H3K9me3	0.06	-0.09	0.04	0.01
H3K9me2	0.04	-0.09	-0.14	0.15
H3K27me3	-0.03	0.13	0.06	-0.34
H3K4me3	-0.02	0.05	0.06	-0.07
H3K36me3	0.00	-0.04	0.14	0.11

log_2 (OIS / G_{ratio}) - log_2(Con_{ratio}) -0.3 0 0.3

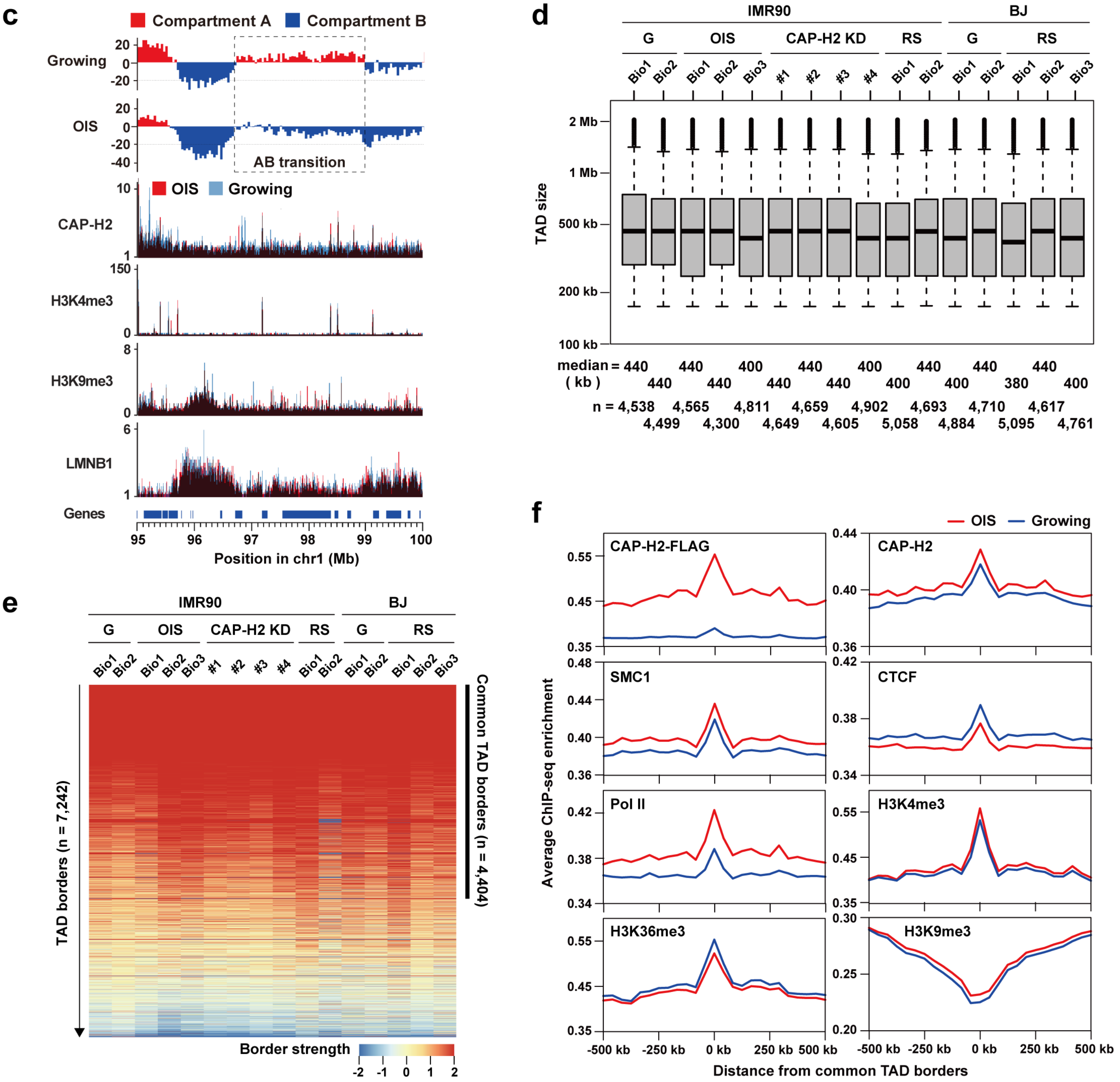

Supplementary Figure 5. Prediction of TADs across all samples and replicates

(**a**) ChIP-seq enrichment of the indicated proteins and epigenetic marks for the respective compartmental categories. Five hundred loci (40 kb bins) were randomly selected from each compartmental category and from the entire human genome (control). Average ChIP-seq enrichment was calculated for each specific category and control, and a ratio of average ChIP-seq enrichment between each category and control was determined. The random sampling was repeated 100 times, and an average $\log_2$ ratio of ChIP-seq enrichment was calculated.
(**b**) Relative ChIP-seq enrichment between OIS and growing cells for the respective compartmental categories. Five hundred loci (40 kb bins) were randomly selected from each compartmental category. Average ChIP-seq enrichment in OIS and growing (G) cells was calculated for each category, and a ratio of the average ChIP-seq enrichment between OIS and G cells was determined. The same calculation was applied to the entire genome (control). These calculations were repeated 100 times, and an average $\log_2$ ratio of ChIP-seq enrichment from the control was subtracted from that from each compartmental category.
(**c**) An example of an AB transition between growing and OIS cells. PCA scores in growing and OIS cells are shown along a 5 Mb region in chromosome 1. ChIP-seq data for CAP-H2, H3K4me3, H3K9me3, and LMNB1 are shown beneath. ChIP-seq data for epigenetic marks and LMNB1 were previously published[2, 5].
(**d**) Size distributions of TADs in growing (G), OIS, CAP-H2 KD and RS (replicative senescence) cells across the entire genome; two different cell lines, IMR90 and foreskin fibroblast BJ, are shown as boxplots (central bar represents the median with boxes indicating the upper and lower quartiles, and whiskers extend to the data points, which are no more than 1.5x the interquartile range from the box, circles show outliers). TADs were predicted using in situ Hi-C data as described in **Methods**. Median sizes and numbers of the predicted TADs are shown at the bottom.
(**e**) A comprehensive list of TAD borders across the genome, obtained by comparing each predicated TAD border to all others. Border strength scores from all the samples were used to calculate an average border strength score for each TAD border (**Methods**). TAD borders were ordered from top to bottom on the basis of average border strength scores. Common TAD borders were defined as average border strength scores > 1.
(**f**) Average ChIP-seq enrichment of the indicated proteins and histone marks at common TAD borders.

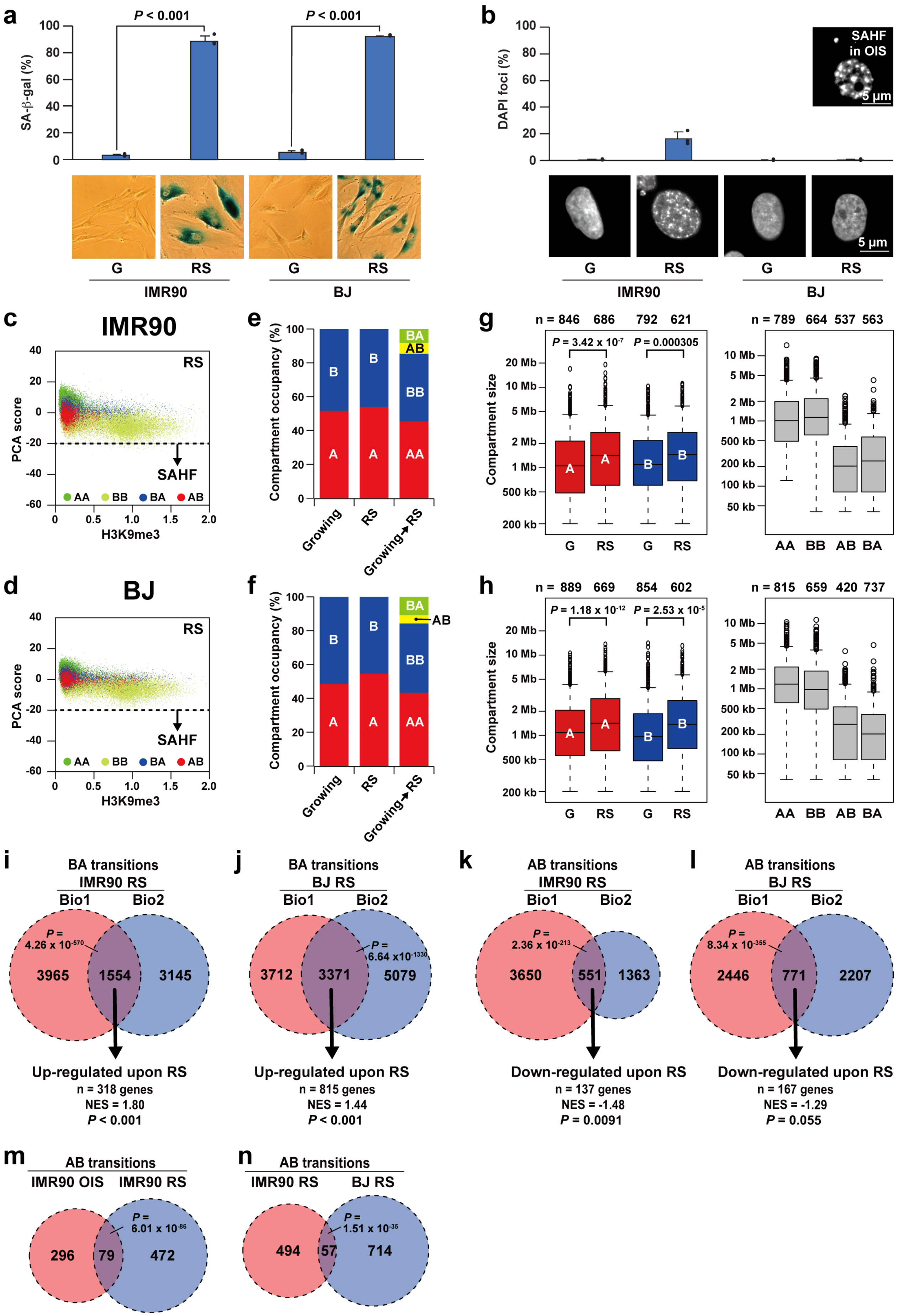
a
P < 0.001
P < 0.001
SA-β-gal (%)
G
RS
G
RS
IMR90
BJ
b
DAPI foci (%)
SAHF in OIS
5 µm
G
RS
G
RS
IMR90
BJ
c
IMR90
PCA score
H3K9me3
RS
SAHF
AA BB BA AB
d
BJ
e
f
Compartment occupancy (%)
Growing
RS
Growing→RS
g
h
Compartment size
n = 846 686 792 621
P = 3.42 x 10⁻⁷
P = 0.000305
n = 789 664 537 563
n = 889 669 854 602
P = 1.18 x 10⁻¹²
P = 2.53 x 10⁻⁵
n = 815 659 420 737
AA BB AB BA
i
BA transitions
IMR90 RS
Bio1 Bio2
P = 4.26 x 10⁻⁵⁷⁰
3965 1554 3145
Up-regulated upon RS
n = 318 genes
NES = 1.80
P < 0.001
j
BA transitions
BJ RS
P = 6.64 x10⁻¹³³⁰
3712 3371 5079
Up-regulated upon RS
n = 815 genes
NES = 1.44
P < 0.001
k
AB transitions
IMR90 RS
P = 2.36 x 10⁻²¹³
3650 551 1363
Down-regulated upon RS
n = 137 genes
NES = -1.48
P = 0.0091
l
AB transitions
BJ RS
P = 8.34 x 10⁻³⁵⁵
2446 771 2207
Down-regulated upon RS
n = 167 genes
NES = -1.29
P = 0.055
m
AB transitions
IMR90 OIS IMR90 RS
P = 6.01 x 10⁻⁸⁶
296 79 472
n
AB transitions
IMR90 RS BJ RS
P = 1.51 x 10⁻³⁵
494 57 714

Supplementary Figure 6. Compartmental reorganizations upon RS

(**a**) SA-β-gal staining of growing and RS cells in the two cell lines (IMR90 and BJ) to detect senescent cells. Cells were prepared as described in **Methods**. *P* values were calculated by two-sided Student's *t* test, using biologically independent samples (n = 3, error bars represent the SD).

(**b**) DAPI staining of growing and RS cells in the same two cell lines as in panel **a**. For comparison, a typical DAPI staining pattern of the nucleus in the IMR90 OIS cell is shown on top; note that DAPI foci in OIS cells had sharper edges and appeared brighter than RS cells.

(**c**) Correlation between PCA score, H3K9me3 enrichment and chromatin compartment for all 40 kb genomic regions across the genome for IMR90 RS cells (Bio1). Dot colors reflect genomic regions belonging to the indicated compartmental categories.

(**d**) Same as panel **c**, but for BJ RS data (Bio1).

(**e**) Occupancy of A and B compartments in IMR90 growing and RS cells. The human genome was divided into 40 kb bins, which were assigned either to A or B compartment. Right column shows the composition of the compartmental categories (AA, BB, AB, and BA) upon switching from growing to RS.

(**f**) Same as panel **e**, but for BJ growing and RS cells.

(**g**) Left: Size distributions of A and B compartments for IMR90 growing and RS cells shown as boxplots (central bar represents the median with boxes indicating the upper and lower quartiles, and whiskers extend to the data points, which are no more than 1.5x the interquartile range from the box, circles show outliers, two-sided Mann–Whitney *U* test). Numbers of A and B compartments are shown at top. Right: Size distributions and numbers of genomic regions that belong to the respective compartmental categories.

(**h**) Same as panel **g**, but for BJ growing and RS cells.

(**i**) Overlap of BA transitions between the IMR90 RS biological replicates (Bio1 and Bio2). Genomic regions (40 kb bins) undergoing BA transitions upon RS were compared between the IMR90 RS biological replicates (hypergeometric distribution test). Genes (n = 318) located at genomic regions undergoing BA transitions that were common between the IMR90 RS biological replicates were subjected to GSEA (bottom) to obtain NES and *P* values.

(**j**) Same as panel **i**, but for the BJ RS replicates (Bio1 and Bio2).

(**k**) Overlap of AB transitions between the IMR90 RS biological replicas (Bio1 and Bio2). Genomic regions (40 kb bins) undergoing AB transitions upon RS were compared between the IMR90 RS biological replicates (hypergeometric distribution test).

(**l**) Same as panel **k**, but for the BJ RS replicates (Bio1 and Bio2).

(**m**) Overlap of AB transitions upon OIS and RS in IMR90 cells. Genomic regions undergoing AB transitions that were common between the IMR90 OIS biological replicates were compared to those shared between the IMR90 RS biological replicates (hypergeometric distribution test).

(**n**) Overlap of AB transitions upon RS in IMR90 and BJ cells. Genomic regions undergoing AB transitions that were common between the IMR90 RS biological replicates were compared to those shared between the BJ RS biological replicates (hypergeometric distribution test).

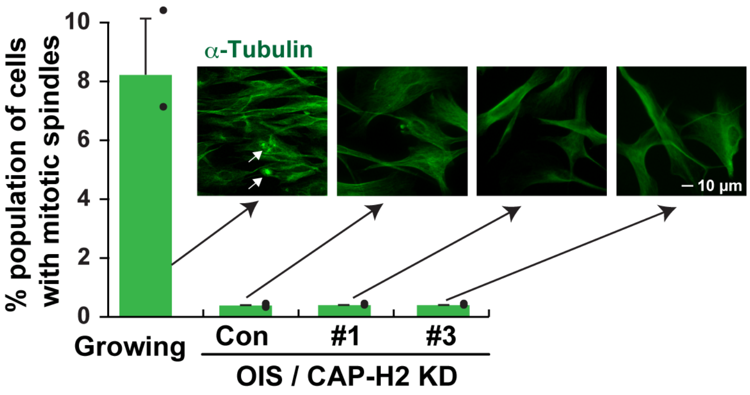

Supplementary Figure 7. Negligible effect of CAP-H2 KD on the cell cycle arrest in OIS cells Cells prepared as in **Fig. 7a** were subjected to IF experiments visualizing spindle microtubules. White arrows indicate cells with mitotic spindles. n = 212, 211 and 308 for growing; n = 247, 281 and 324 for Con; n = 254, 250 and 318 for shRNA #1; n = 255, 264 and 302 for shRNA #3. *P* values were calculated by two-sided Student's *t* test, using biologically independent samples (n = 3, error bars represent the SD).

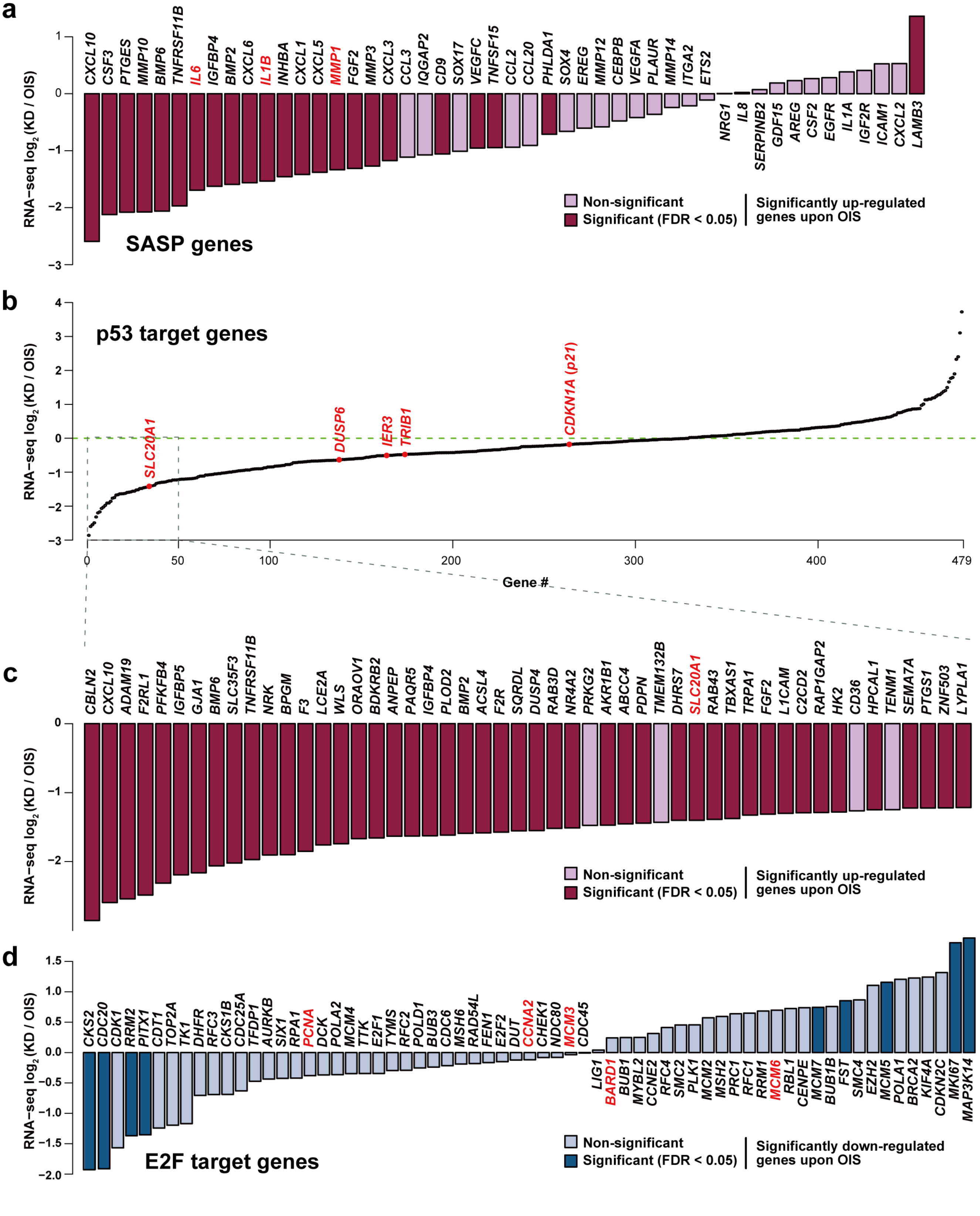

Supplementary Figure 8. SASP and p53 target genes were frequently down-regulated by CAP-H2 KD

(**a**) SASP genes significantly up-regulated in OIS cells compared to growing cells were ranked by expression ratios between CAP-H2 KD and OIS cells. OIS cells were subjected to CAP-H2 KD. Significantly up- and down-regulated genes in CAP-H2 KD cells compared to OIS cells were indicated by darker color. Genes in red color were subjected to RT-qPCR analysis (**Fig. 8**).
(**b**) Same as panel **a**, but p53 target genes were subjected to the analysis.
(**c**) Top 50 genes ranked in panel **b** were enlarged.
(**d**) E2F target genes significantly down-regulated in OIS cells compared to growing cells were ranked by expression ratios between CAP-H2 KD and OIS cells. Significantly up- and down-regulated genes in CAP-H2 KD cells compared to OIS cells were indicated by darker color.

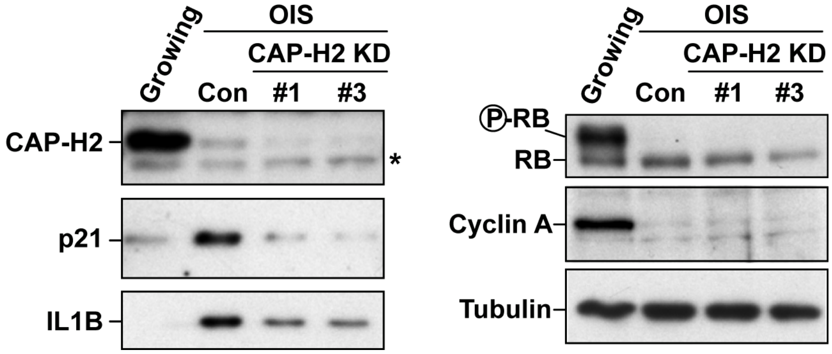

Supplementary Figure 9. Reduction of p21 and IL1B protein levels by CAP-H2 KD (left)
Cells prepared as in **Fig. 7a** were subjected to western blotting using the indicated antibodies. OIS cells were infected with lentivirus encoding one of the two shRNA constructs (#1 and #3) against CAP-H2 gene (*NCAPH2*) or carrying an empty vector (control) and harvested 3 days after the lentivirus infection. Asterisk indicates non-specific band. The same analysis was performed to detect RB and Cyclin A (right).

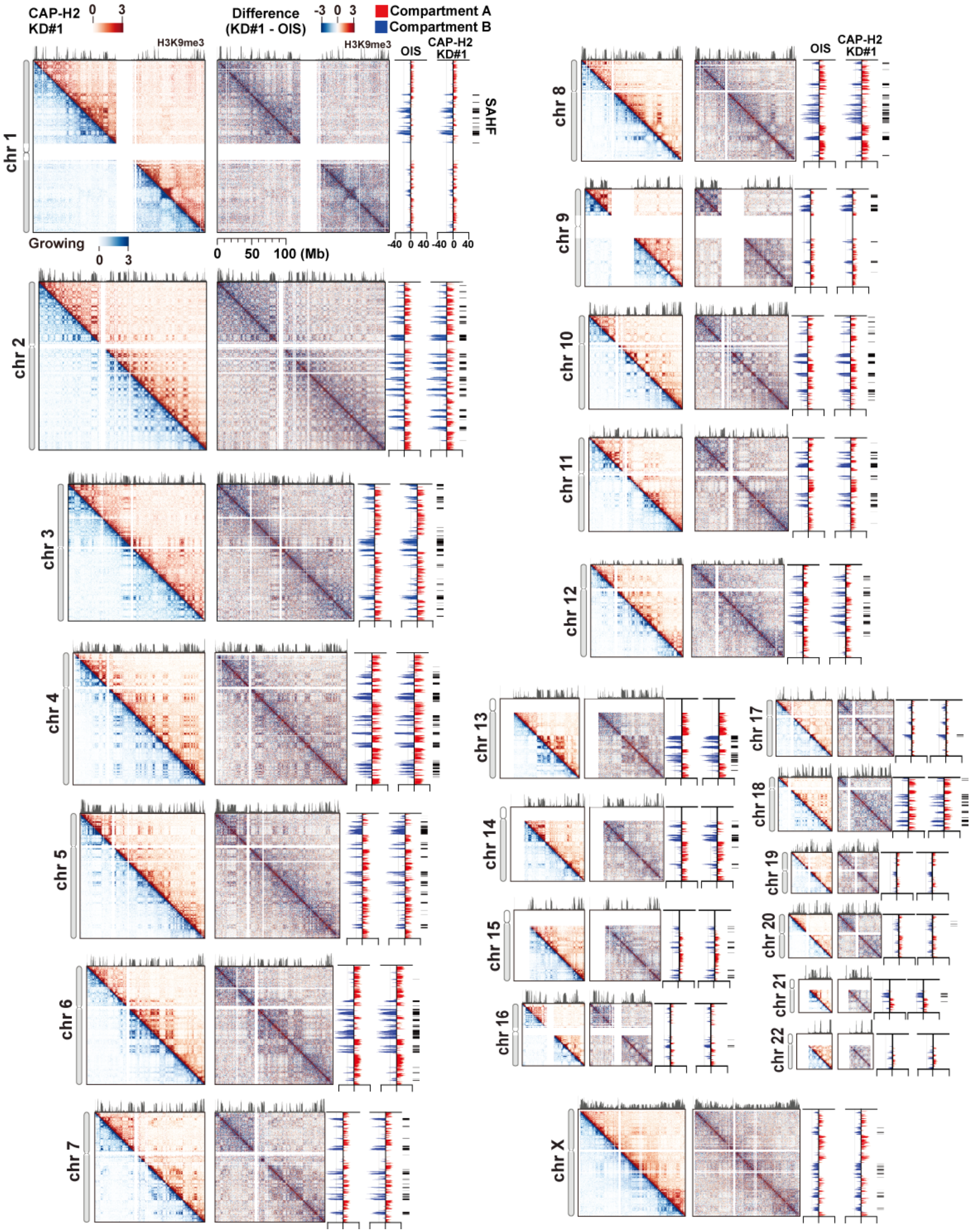
CAP-H2 KD#1
0 3
Difference (KD#1 - OIS)
-3 0 3
Compartment A
Compartment B
H3K9me3
OIS
CAP-H2 KD#1
SAHF
Growing
0 3
0 50 100 (Mb)
-40 0 40 -40 0 40
chr 1
chr 2
chr 3
chr 4
chr 5
chr 6
chr 7
chr 8
chr 9
chr 10
chr 11
chr 12
chr 13
chr 14
chr 15
chr 16
chr 17
chr 18
chr 19
chr 20
chr 21
chr 22
chr X

Supplementary Figure 10. Genome-wide contact maps at 200 kb resolution in CAP-H2 KD#1 (top right) and OIS (bottom left) cells

Cells were prepared as described in **Fig. 7a**. Histone H3K9me3 ChIP-seq data (GEO accession#, GSE38448[https://www.ncbi.nlm.nih.gov/geo/query/acc.cgi?acc=GSE38448]) in growing IMR90 cells are shown on top. Difference of contact probabilities between CAP-H2 KD#1 and OIS cells are also shown as described in **Fig. 9b** (middle). Red and blue dots indicate that contact probabilities were higher in CAP-H2 KD#1 and OIS cells, respectively. PCA scores in CAP-H2 KD#1 and OIS cells were plotted along chromosomes (right). SAHF were defined as genomic regions with PCA scores below -20 and annotated as black bars along chromosomes.

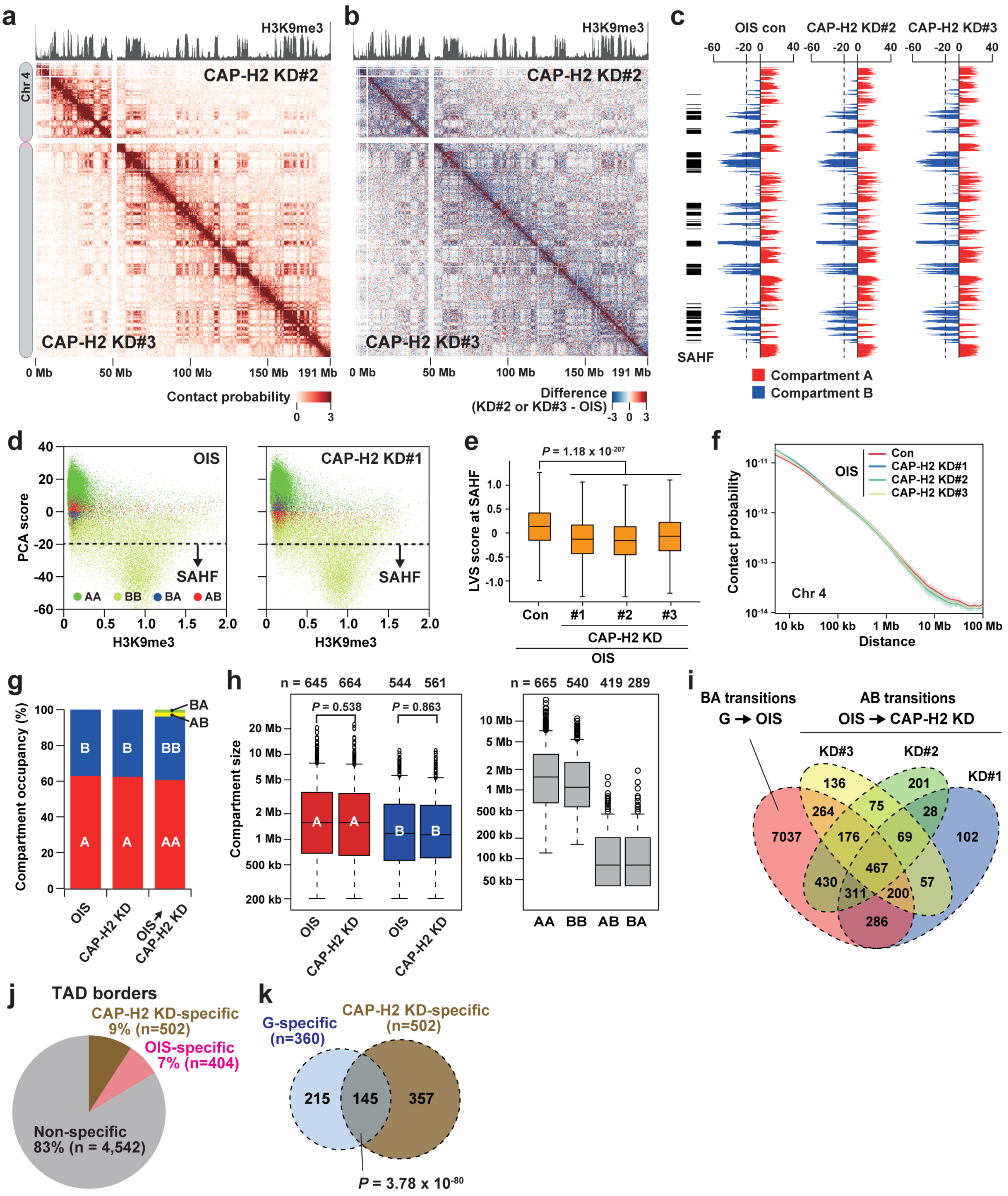

Supplementary Figure 11. Effects of condensin depletion on the compartmental organization

(**a**) Contact maps for chromosome 4 after KD of CAP-H2 in OIS cells, at 200 kb resolution; CAP-H2 KD#2 (top right) and CAP-H2 KD#3 (bottom left). Histone H3K9me3 ChIP-seq data (GEO accession#, GSE38448[https://www.ncbi.nlm.nih.gov/geo/query/acc.cgi?acc=GSE38448]) are shown at top. CAP-H2 KD (IMR90) cells were prepared as in **Fig. 7a**. All panels in this figure used data from the IMR90 cell line.

(**b**) Difference of contact probabilities between OIS cells and either CAP-H2 KD#2 (top right) or KD#3 (bottom left) cells. Red and blue dots indicate that contact probabilities were higher in CAP-H2 KD (#2 and #3) and OIS cells, respectively.

(**c**) PCA scores in OIS and CAP-H2 KD (#2 and #3) cells plotted along chromosome 4. SAHF were defined as genomic regions with PCA scores < -20 in OIS cells and annotated as black bars along the chromosome (left).

(**d**) Degree of histone H3K9me3 enrichment at various PCA scores, for every 40 kb bin across the human genome, in OIS and CAP-H2 KD (#1) cells. Dot colors reflect genomic regions belonging to the indicated compartmental categories.

(**e**) Distributions of LVS scores at SAHF regions in OIS and CAP-H2 KD (#1–#3) cells shown as boxplots (central bar represents the median with boxes indicating the upper and lower quartiles, and whiskers extend to the data points, which are no more than 1.5x the interquartile range from the box, two-sided Mann–Whitney *U* test). SAHF regions were defined by PCA scores < -20 in OIS cells. Distributions of LVS scores within SAHF regions were summarized as boxplots.

(**f**) Relations between contact probabilities and genomic distances in the OIS and CAP-H2 KD (#1–#3) data. Distance curves were generated using contact probabilities at 100 bp resolution.

(**g)** Occupancy of A and B compartments in OIS and CAP-H2 KD#1 cells. The human genome was divided into 40 kb bins, which were assigned to either A or B compartment. Rightmost column shows the composition of the compartmental categories (AA, BB, AB, and BA) upon CAP-H2 KD in OIS cells.

(**h**) Left: Size distributions of A and B compartments for OIS and CAP-H2 KD#1 cells shown as boxplots (central bar represents the median with boxes indicating the upper and lower quartiles, and whiskers extend to the data points, which are no more than 1.5x the interquartile range from the box, outliers shown as circles, two-sided Mann–Whitney *U* test). Numbers of A and B compartments are shown at top. Right: Size distributions and numbers of genomic regions that belong to the respective compartmental categories.

(**i**) Overlap of genomic regions undergoing BA transitions upon OIS and AB transitions upon CAP-H2 KD (#1–#3) in OIS cells. Genomic regions (40 kb bins) undergoing BA transitions upon OIS were compared with those undergoing AB transitions by CAP-H2 KD in OIS cells.

(**j**) Numbers of TAD borders that were specific to OIS and CAP-H2 KD cells. TAD borders were predicted from the OIS (Bio1–3) and CAP-H2 KD (#1–#3) data. TAD borders conserved in at least two OIS replicates but not in CAP-H2 KD data were classified as OIS-specific borders; TAD borders conserved in at least two CAP-H2 KD data but not in OIS data were categorized as CAP-H2 KD-specific borders. Remaining TAD borders detected in at least two data sets were classified as non-specific borders.

(**k**) Significant overlap between CAP-H2 KD-specific borders and G-specific borders as predicted in **Fig. 5a** (hypergeometric distribution test).

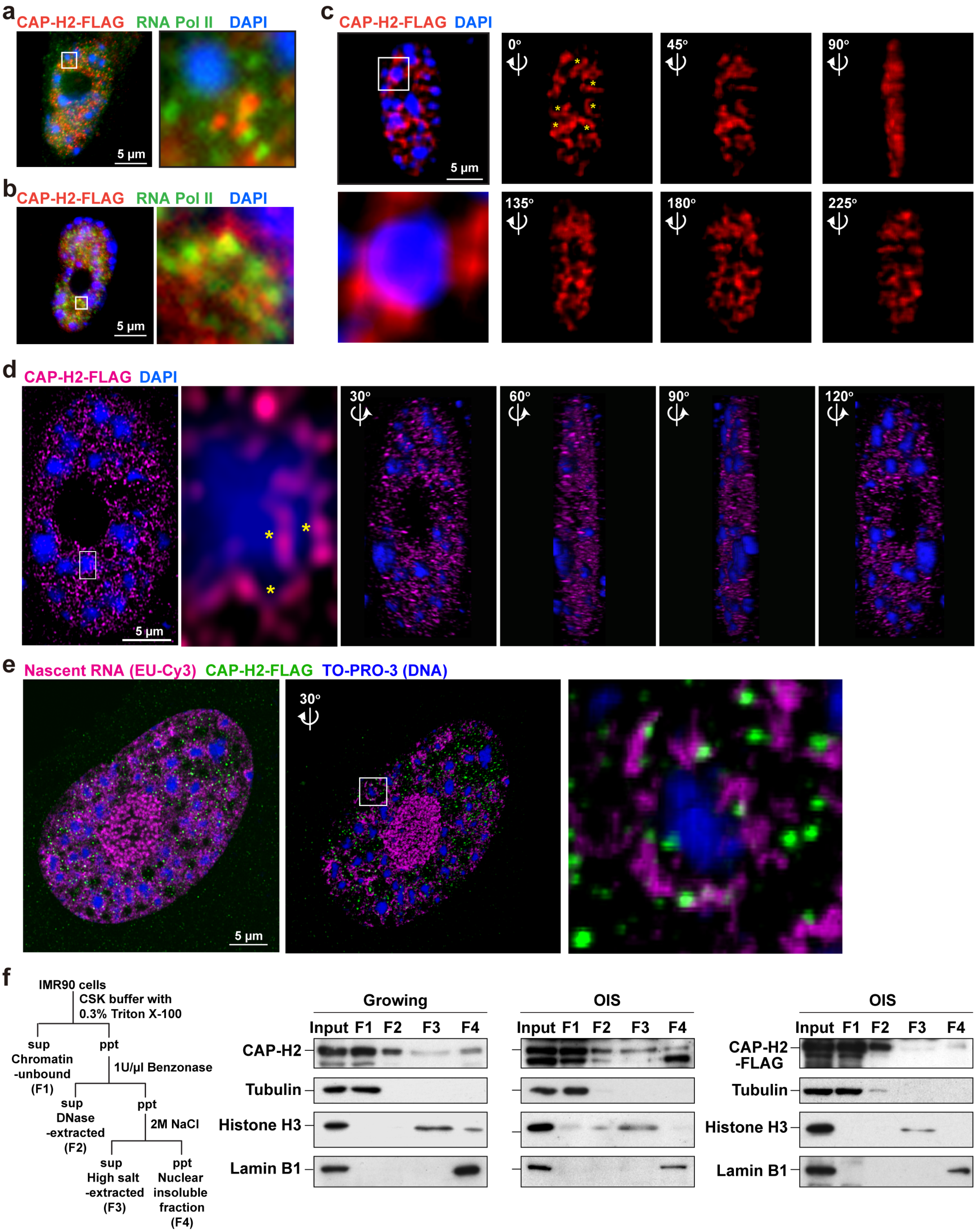

Supplementary Figure 12. Nuclear localization of CAP-H2 condensin II subunit in OIS cells

(**a**) Immunofluorescent visualization of CAP-H2-FLAG and RNA Pol II in IMR90 OIS cells using mouse monoclonal anti-FLAG (Sigma Aldrich, clone M2) and rabbit polyclonal anti-Pol II (Bethyl Laboratories, A300-653A) antibodies.

(**b**) Immunofluorescent visualization of CAP-H2-FLAG and RNA Pol II in IMR90 OIS cells using rabbit polyclonal anti-Myc (Abcam, ab9106) and mouse monoclonal anti-Pol II (Covance, 8WG16) antibodies. Note that CAP-H2-FLAG proteins also consist of the secondary MYC epitope. These images were captured using Zeiss Axioimager Z1 fluorescence microscope.

(**c**) Upper left: CAP-H2-FLAG proteins visualized in OIS cells were detected using a Leica SP5 II laser scanning confocal microscope. The images were acquired at 0.2-μm intervals in the z axis controlled by LAS AF software (Leica Microsystems). Lower left: Enlarged view of the indicated area. Right: Three-dimensional, rotated images constructed using ImageJ 3D viewer plugin (http://imagej.nih.gov/). Asterisks indicate potential filamentous distributions of CAP-H2 proteins.

(**d**) Left: CAP-H2-Flag proteins were detected using Zeiss Airyscan super-resolution microscope. 2nd Left: Enlarged view of the indicated area. Asterisks indicate potential filamentous distributions of CAP-H2 proteins. Right: Three-dimensional, rotated images constructed using Imaris v. 9.2.

(**e**) Left: CAP-H2-Flag proteins and EU-incorporated RNA were co-visualized in OIS cells, and the image was captured by Zeiss Airyscan super-resolution microscope. RNA visualization was performed as in **Supplementary Fig. 2k** (**Supplementary Methods**). Middle: Three-dimensional rotated image. Right: Enlarged view of the indicated area.

(**f**) Left: Fractionation process for OIS and growing cells (**Supplementary Methods**). Right: Western blot of the indicated proteins in the respective fractions (F1, F2, F3 and F4).

Supplementary Tables

Supplementary Table 1. Read numbers remaining after the respective filtering processes

Filtering processes	IMR90_G_Bio1		IMR90_G_Bio2		IMR90_OIS_Bio1	
Sequenced reads	86,305,544	100%	123,532,180	100%	64,143,603	100%
Both sides aligned	85,577,267	99.2%	122,574,821	99.2%	63,333,577	98.7%
PCR bias removed	68,950,991	79.9%	119,997,744	97.1%	60,458,787	94.3%
Excluding repeat-derived reads	40,114,222	46.5%	73,690,392	59.7%	35,203,728	54.9%
Phread quality > 30	33,294,433	38.6%	54,546,495	44.2%	29,043,500	45.3%
(Potential self-ligation reads)	32,130	0.04%	33,897	0.03%	486,370	0.76%
(Potential undigested reads)	1,971,784	2.28%	7,138,425	5.78%	3,576,641	5.58%
Inter-chromosome	9,517,084	11.0%	14,268,232	11.6%	10,024,284	15.6%
Intra-chromosome	21,773,353	25.2%	33,105,799	26.8%	14,956,148	23.3%
Map resolution (bp) *	21,950		16,750		28,600	

Filtering processes	IMR90_OIS_Bio2		IMR90_OIS_Bio3		IMR90_OIS_CAPH2_KD#1	
Sequenced reads	144,898,104	100%	105,032,852	100%	67,871,333	100%
Both aligned	143,808,970	99.2%	103,587,839	98.6%	66,971,641	98.7%
PCR bias removed	140,575,527	97.0%	100,977,152	96.1%	64,215,508	94.6%
Excluding repeat-derived reads	87,150,710	60.1%	58,601,265	55.8%	37,281,686	54.9%
Phread quality > 30	65,953,436	45.5%	43,896,761	41.8%	30,374,151	44.8%
(Potential self-ligation reads)	181,569	0.13%	3,760	0.00%	604,169	0.89%
(Potential undigested reads)	7,671,747	5.29%	5,322,598	5.07%	2,615,951	3.85%
Inter-chromosome	16,862,885	11.6%	7,527,661	7.2%	10,612,144	15.6%
Intra-chromosome	41,237,016	28.5%	31,042,599	29.6%	16,541,804	24.4%
Map resolution (bp) *	13,600		20,100		27,050	

Filtering processes	IMR90_OIS_CAPH2_KD#2		IMR90_OIS_CAPH2_KD#3		IMR90_OIS_CAPH2_KD#4	
Sequenced reads	67,540,806	100%	67,032,805	100%	105,671,013	100%
Both aligned	66,753,844	98.8%	66,211,953	98.8%	103,940,734	98.4%
PCR bias removed	64,405,722	95.4%	63,573,367	94.8%	101,465,182	96.0%
Excluding repeat-derived reads	37,224,266	55.1%	36,800,996	54.9%	59,210,631	56.0%
Phread quality > 30	30,092,498	44.6%	29,588,011	44.1%	44,847,555	42.4%
(Potential self-ligation reads)	409,450	0.61%	470,142	0.70%	0	0.00%
(Potential undigested reads)	3,472,103	5.14%	2,613,435	3.90%	6,818,616	6.45%
Inter-chromosome	10,877,601	16.1%	10,217,040	15.2%	7,849,409	7.4%
Intra-chromosome	15,333,291	22.7%	16,287,349	24.3%	30,179,382	28.6%
Map resolution (bp) *	27,100		27,600		19,350	

Filtering processes	IMR90_RS_Bio1		IMR90_RS_Bio2		BJ_G_Bio1	
Sequenced reads	82,003,987	100%	64,597,967	100%	62,117,403	100%
Both aligned	81,175,312	99.0%	63,974,976	99.0%	61,664,849	99.3%
PCR bias removed	78,686,317	96.0%	35,605,191	55.1%	56,834,548	91.5%
Excluding repeat-derived reads	45,524,903	55.5%	18,674,328	28.9%	34,400,819	55.4%
Phread quality > 30	36,837,824	44.9%	14,891,449	23.1%	28,918,824	46.6%
(Potential self-ligation reads)	38,500	0.05%	84,648	0.13%	57,031	0.09%
(Potential undigested reads)	1,790,229	2.18%	738,415	1.14%	1,624,562	2.62%
Inter-chromosome	10,647,560	13.0%	4,474,664	6.9%	6,700,376	10.8%
Intra-chromosome	24,361,431	29.7%	9,593,682	14.9%	20,536,770	33.1%
Map resolution (bp) *	22,850		28,750		32,200	

Supplementary Table 1 (*continued*)

Filtering processes	BJ_G_Bio2		BJ_RS_Bio1		BJ_RS_Bio2	
**Sequenced reads**	103,526,587	100%	60,969,142	100%	86,721,565	100%
**Both aligned**	102,132,469	98.7%	60,511,079	99.2%	85,818,635	99.0%
**PCR bias removed**	99,654,528	96.3%	58,509,581	96.0%	83,426,523	96.2%
**Excluding repeat-derived reads**	56,770,427	54.8%	34,969,646	57.4%	48,186,231	55.6%
**Phread quality > 30**	41,767,094	40.3%	29,367,778	48.2%	37,954,569	43.8%
**(Potential self-ligation reads)**	0	0.00%	93,115	0.15%	257,536	0.30%
**(Potential undigested reads)**	2,372,984	2.29%	1,986,624	3.26%	1,660,565	1.91%
**Inter-chromosome**	7,907,116	7.6%	8,573,557	14.1%	11,969,636	13.8%
**Intra-chromosome**	31,486,844	30.4%	18,714,390	30.7%	24,066,747	27.8%
**Map resolution (bp) ***	20,300		30,250		21,400	

Filtering processes	BJ_RS_Bio3	
**Sequenced reads**	108,216,968	100%
**Both aligned**	106,769,029	98.7%
**PCR bias removed**	104,316,074	96.4%
**Excluding repeat-derived reads**	59,289,163	54.8%
**Phread quality > 30**	43,853,503	40.5%
**(Potential self-ligation reads)**	0	0.00%
**(Potential undigested reads)**	2,338,395	2.16%
**Inter-chromosome**	8,937,792	8.3%
**Intra-chromosome**	32,577,189	30.1%
**Map resolution (bp) ***	19,500	

*The highest map resolutions were defined as previously described[6].

Supplementary Table 2. Primers used for ChIP-qPCR

Gene	Forward primer (5' - 3')	Reverse primer (5' - 3')
p21	TGTGGCTCTGATTGGCTTTC	AGCCCAAGGACAAAATAGCC
IER3	GCCTGGTGTTTCTTTGTGGT	CTGGACCTCAGCACTTTCCT
TRIB1	GCTGGAGACTCATCGCTTTG	CCGCGAGGACAGGATTCAG

Supplementary Table 3. Primers used for RT-qPCR

Gene	Forward primer (5' - 3')	Reverse primer (5' - 3')
IL1B	AGCTCGCCAGTGAAATGATGG	GTCCTGGAAGGAGCACTTCAT
Il6	ACATCCTCGACGGCATCTCA	TCACCAGGCAAGTCTCCTCA
B2M	GGCATTCCTGAAGCTGACA	CTTCAATGTCGGATGGATGAAAC
MMP1	AATAGTGGCCCAGTGGTTGA	GGCTGCTTCATCACCTTCAG
CCL20	CTCCTGGCTGCTTTGATGTC	TGCTTGCTGCTTCTGATTCG
p21	CCTGTCACTGTCTTGTACCCT	GCGTTTGGAGTGGTAGAAATCT
DUSP6	GTTGATGGCCGACTCGATG	GGAAATGGCGATCAGCAAGA
SLC20A	ATGCTCATCCTGGGCTTCAT	GCTTCAGGGTCACTACACCT
IER3	GCCTGGTGTTTCTTTGTGGT	CTGGACCTCAGCACTTTCCT
TRIB1	GCTGGAGACTCATCGCTTTG	CCGCGAGGACAGGATTCAG
Cyclin A	CTGCATTTGGCTGTGAACTAC	ACAAACTCTGCTACTTCTGGG
MCM3	AAAGTCGTCCGCAGTGTCCA	CGGCATCTCCTGGATGGTGA
MCM6	CCAGGAGCACGTGCAGAAAC	CGCAACACAGCAGGCAAGAA
PCNA	CAACGAGGCCTGCTGGGATA	TCTTCATTGCCGGCGCATTT
BARD1	GAAGAGCTTGGCCGGTTTCG	AAGCAAGGAAGCCTCGGGAA
NCAPH2	AGGAGCAGCCAATGGAAGTT	AGCTCTACTGCCTCCTCTGC

Supplementary Notes

Characterization of CAP-H2 antibodies

A condensin II-specific subunit, CAP-H2, was detected by CAP-H2 antibody and depleted using the three shRNA constructs (#1, #2, and #3; **Supplementary Fig. 1a,b**). *NCAPH2* mRNA were more abundant in growing cells than OIS cells (**Supplementary Fig. 1c**). We tested two different CAP-H2 antibodies and found that CAP-H2 proteins were detected by the both antibodies (Bethyl Laboratories, A302-275A; Abgent, AP1973A) and were much decreased by CAP-H2 KD (**Supplementary Fig. 1d**). CAP-H2 detected by the Bethyl antibody was reduced after induction of OIS, whereas CAP-H2 detected by the Abgent antibody was enhanced, potentially suggesting that the Abgent antibody might recognize modifications of CAP-H2 proteins (**Supplementary Fig. 1d**). Furthermore, the Bethyl CAP-H2 antibody detected exogenous CAP-H2-FLAG proteins, again indicating that this antibody recognizes CAP-H2 proteins (**Supplementary Fig. 1e**). We also performed cellular fractionation analysis as previously described[1] and found that CAP-H2 interacting with chromatin was clearly detected by the Bethyl antibody (**Supplementary Fig. 1f,g**). Therefore, we employed the Bethyl CAP-H2 antibody to map condensin II across the human genome.

Definition of condensin II binding sites in OIS cells

For OIS cells, binding peaks of endogenous CAP-H2 and exogenous CAP-H2-FLAG proteins were determined by ChIP-seq. ChIP-seq experiments were performed twice for the respective proteins (biological replicas): CAP-H2-FLAG (OIS#1), CAP-H2-FLAG (OIS#2), CAP-H2 (Bethyl 275 OIS#1), and CAP-H2 (Bethyl 275 OIS#2). CAP-H2-FLAG binding peaks conserved between two biological replicas were obtained. Subsequently, the CAP-H2-FLAG common peaks were compared to endogenous CAP-H2 peaks. If the CAP-H2-FLAG common peaks were overlapped with the endogenous CAP-H2 peaks in at least one of biological replicas, then they were defined as CAP-H2 binding sites (**Supplementary Fig. 2c**). For growing cells, binding peaks of endogenous CAP-H2 (Bethyl 275 G) and exogenous CAP-H2-FLAG (G) were determined by performing ChIP-seq experiments once for the respective proteins, and their common peaks were defined as CAP-H2 binding sites.

Classification of ChIP-seq binding sites

Transcriptional start sites (TSSs) of protein-coding genes were selected, and less than 1 kb regions upstream and downstream from TSSs were defined as TSS sections. Histone H3K4me3

peaks were previously mapped in OIS and growing cells[2]. TSS sections overlapped with H3K4me3 peaks were defined as active promoters, and others were classified as inactive promoters.

Non-coding RNA genes were defined as follows: Locations of tRNA genes were annotated at GtRNAdb (http://gtrnadb.ucsc.edu/genomes/eukaryota/Hsapi19/); From Ensembl database (https://useast.ensembl.org), lincRNAs, miRNAs, miscRNAs, snRNAs, snoRNAs and rRNAs were selected. Genomic regions encoding these genetic elements were defined as non-coding RNA genes.

Definition of potential enhancers (for Fig. 1)

Enhancers were defined based on histone H3K27ac peaks in OIS and growing cells[7]. H3K27ac peaks were determined by the parameters: FDRs < 0.01, P values < 0.0001, and fold enrichment > 4. All TSSs with H3K27ac peaks were eliminated, and remaining H3K27ac peaks were defined as potential enhancers. Super enhancers were defined using HOMER software using the option "-style super". Enhancers not overlapped with super enhancers were defined as typical enhancers.

Supplementary Methods

Treatment of human cells with RNA polymerase inhibitors

RNA polymerase II inhibitors, Triptolide and α-Amanitin, were used to impair transcription in human cells, as described previously[8]. Final 1 μM Triptolide (Sigma Aldrich, T3652) or 5 μg/ml α-Amanitin (Sigma Aldrich, A2263) was added to culture medium, and cells were cultured for the indicated duration.

Calculation of LVS scores

LVS scores were calculated from ICE normalized contact matrixes at 40 kb resolution, as previously described (SVL scores)[9]. LVS scores were defined as follows:

LVS scores = (Sum of contact probabilities for genomic combinations between 2 and 50 Mb) ÷ (Sum of contact probabilities for genomic combinations within 2 Mb), (1)

where contact probabilities for combinations within 40 kb (= intra-bins along diagonal lines) were eliminated from the calculation.

Visualization of nascent RNA using 5-ethynyl uridine (EU)

Nascent RNA was monitored using EU as detailed previously[10]. Final 0.5 mM 5-ethynyl uridine (EU) (Click Chemistry Tools) was added to medium and cultured for 1 hour. EU-incorporated transcripts were visualized by utilizing reactivity between EU and azide conjugated with Cy3. Cells grown on coverslip were fixed with 1% pFA in PBS containing 200 mM sucrose at room temperature for 10 minutes. After washing cells three times with 1 ml PBS, cells were permeabilized with 0.5% Triton-X100 in PBS at 4ºC for 5 minutes and washed three time with 1 ml TBS (20 mM Tris-HCl pH 7.5, 150 mM NaCl). "Click" reaction that connects EU and azide was performed at room temperature for 30 minutes in dark using Click buffer 1 [100 mM Tris-HCl pH 8.0, 1 mM $CuSO_4$, 100 mM ascorbic acid, 10 μM Cy3-azide (Click Chemistry Tools)]. After washing cells three times with 1 ml PBST (PBS containing 0.05% Tween 20), genomic DNA was stained with 10 μg/ml DAPI in PBS at room temperature for 10 minutes. After washing cells twice with 1 ml PBST, the coverslip was mounted onto slide glass using ProLong Gold antifade mountant (Thermo Fisher Scientific).

To quantify Cy3 signals in the nucleus, low magnified images were captured by Nikon 80i Upright Microscope with 20 x objective lens. Nuclear area was defined by DAPI signals, and total Cy3 signals were quantified for each nucleus using NIS-Elements Microscope Imaging Software (Nikon). As a negative control, cells were cultured without EU and subjected to the same procedure. Cy3 signals derived from cells not treated by EU were quantified and used as background. Nascent RNA was calculated as follows.

Total nascent RNA per nucleus = Total Cy3 signals in selected nuclear area – (Nuclear area x *Average background signal density),* (2)

where nuclear area was estimated by the number of pixels with DAPI staining. Average background signal density was calculated by total nuclear Cy3 signals divided by nuclear area (pixels) in negative control cells, as described above. Distributions of total nascent RNA per nucleus were plotted as boxplots for respective conditions and subjected to two-sided Mann–Whitney *U* test.

Co-visualization of nascent RNA and CAP-H2

Final 0.5 mM EU was added to medium and cultured for 1 hour. To co-visualize nascent RNA and CAP-H2 proteins, cells grown on coverslip were fixed with 1% pFA in 1 ml PBS containing 200 mM sucrose at room temperature for 10 minutes. After washing cells three times with 1 ml PBS, cells were permeabilized with 0.5% Triton-X100 in 1 ml PBS at 4ºC for 5 minutes and washed three times with 1 ml PBST. Cells were soaked in PBS containing 1% BSA at room temperature for 10 minutes and incubated with 1:1000-diluted mouse monoclonal anti-FLAG M2 (Sigma Aldrich) in PBS for 30 minutes. After washing cells three times with 1 ml PBST, cells were incubated with 1:1000-diluted Alexa Flour 488-conjugated anti-mouse IgG (Thermo Fisher Scientific) in PBS for 15 minutes. After washing cells three times with TBS buffer, cells on coverslip were subsequently subjected to Click reaction to visualize nascent RNA as described above. Genomic DNA was stained with 1 µM TO-PRO-3 (Thermo Fisher Scientific) in PBS at room temperature for 10 minutes. After washing cells twice with 1 ml PBST, the coverslip was mounted onto slide glass using ProLong Gold antifade mountant (Thermo Fisher Scientific).

Quantification of EU-incorporated nascent RNA by RT-qPCR and RNA-seq

Isolation of nascent RNA was performed as described previously[11] with slight modifications. Final 0.5 mM EU was added to medium and cultured for 1 hour. Total RNA was purified from EU-treated cells using RNeasy mini kit (Qiagen). Click reaction was performed at 45ºC for 30 minutes in dark by incubating RNA with Click buffer 2 [100 mM Tris-HCl pH 7.5, 10% acetonitrile, 15 mM ascorbic acid, 100 mM PMDTA, 1 mM $CuSO_4$, 0.5 mM biotin-azide (Click Chemistry Tools)]. Please note that EU-incorporated RNA was coupled to biotin-azide in this experiment. RNA was purified by ethanol precipitation and dissolved in 50 μl TNE 0.2 buffer (10 mM Tris-HCl pH 7.5, 1 mM EDTA, 0.2 M NaCl). RNA was denatured at 65ºC for 5 minutes and immediately chilled on ice. Biotinylated RNA was pulled down by incubating RNA with MyOne Streptavidin T1 Dynabeads (Thermo Fisher Scientific) at 18ºC for 20 minutes in dark. Magnetic beads were washed twice with 500 μl TNE 2.0 buffer (10 mM Tris 7.5, 1 mM EDTA, 2 M NaCl), twice with 500 μl Wash buffer (100 mM Tris 7.5, 1 mM EDTA, 1 M NaCl, 0.1% Tween 20) with incubation at 65ºC for 10 minutes, and twice with 500 μl TNE 0.2 buffer. RNA-coupled beads were suspended in 10 μl nuclease-free water and subjected to reverse transcription using High-Capacity cDNA Reverse Transcription kit (Thermo Fisher Scientific). cDNA was used as a template in subsequent qPCR with gene specific primers (Power SYBR Green master mix, Thermo Fisher scientific). In order to normalize cell numbers in respective samples, genomic DNA was quantified using total RNA as a template and *p21* primers for qPCR (**Supplementary Table 2**).

To make nascent RNA-seq libraries, cDNA generated from streptavidin bead-bound RNA was subjected to second strand synthesis using NEBNext Ultra II Non-Directional RNA Second Strand Synthesis Module (New England Biolabs). Double-stranded DNA was purified using SPRI beads (Beckman Coulter), followed by library preparation using NEBNext Ultra II DNA Library Prep Kit for Illumina (New England Biolabs). Adaptor-ligated DNA fragments were amplified for 10 cycles using NEBNext Q5 Hot Start HiFi PCR master Mix and NEBNext multiplex oligos (New England Biolabs). Nascent RNA-seq libraries were sequenced on Illumina NextSeq 500 platform to obtain 75-bp single-end reads. Nascent RNA-seq data were obtained for OIS cells treated with either Triptolide for 5 minutes or α-Amanitin for 60 minutes and also for cells without RNA polymerase inhibitor treatment (control) and processed as described in the RNA-seq section. Moreover, nascent RNA levels of *CCL20*, *MMP1*, *IER3*, and *TRIB1* genes were quantified by RT-qPCR using the same nascent RNA samples. Please note that nascent RNA-seq data were strongly correlated with the RT-qPCR results: coefficient of determination R^2 = 0.984 for Triptolide (5 minutes), R^2 = 0.904 for α-Amanitin (60 minutes), and R^2 = 0.987 for no inhibitor treatment.

Therefore, nascent RNA-seq data were normalized by the linear regression slopes obtained from comparison with the RT-qPCR results.

Confocal microscopy

Confocal images were captured using a Leica TCS SP5 II AOBS spectral laser scanning confocal microscope (Leica Microsystems, Inc., Buffalo Grove, IL) mounted on a DMI6000 inverted microscope. The system includes a 405 nm diode, multi-line Argon, and 561 nm DPSS laser with three standard PMTs and two Leica HyD detectors. Images were captured at 1400 Hz with a 63 × HCX PL APO CS oil immersion objective (NA = 1.4). Multi-channel image stacks were acquired at each Z point. Construction of 3D images was carried out using ImageJ 3D viewer plugin (http://imagej.nih.gov/).

Super-resolution microscopy

Airyscan images were acquired on a Zeiss LSM 880 with Airyscan running ZEN v. 2.3 (Carl Zeiss Microscopy Gmbh, Jena). The stand was an inverted Axio Observer, and the objective lens used for all Airyscan images was a Zeiss 63x/1.4 NA Plan Apochromat oil immersion lens. Three-color images were acquired using the 488 nm line of an argon laser (for excitation of AlexaFluor 488), the 561 DPSS laser (for excitation of Cy3), and a 633 nm HeNe laser for excitation of TO-PRO-3. Two-color images were acquired using a 405 nm diode laser for excitation of DAPI and a 561 nm DPSS laser for excitation of Cy3. When acquiring raw Airyscan data, the pixel dwell time was set between 1 and 2 microseconds, while pixel size was always set to 44 nanometers and a step size of 160 nm was used for z-stack acquisition. Bit depth for the raw image data was 8 bits per pixel. 3D Airyscan processing was done using ZEN 2.3, and "Auto" filter strength was always chosen. Rendering of processed 3D data to generate views from different angles was done using Imaris v. 9.2 (Bitplane, Oxford Instruments, Zurich).

Fractionation analysis

Fractionation analysis was performed as previously described[12] with slight modifications. Cells were lysed at 4°C for 15 minutes with 400 µl CSK buffer [10 mM PIPES pH 6.8, 100 mM NaCl, 300 mM sucrose, 3 mM $MgCl_2$, 1 mM EGTA, 1 mM DTT, 0.25 mM PMSF, 10 mM NaF, 0.1 mM ATP, Complete protease inhibitor cocktail (Roche)] containing 0.3% Triton X-100. Soluble fraction (fraction 1, chromatin-unbound fraction) was separated by centrifugation at 3,000 rpm for 3 minutes. The pellet was suspended with 400 µl CSK buffer and incubated with 400 units of

Bezonase nuclease (Sigma Aldrich) at 4ºC for 30 minutes. After centrifugation at 5,000 rpm for 3 minutes, the supernatant was recovered as fraction 2 (DNase-extractable fraction). The pellet was suspended in 400 µl CSK buffer containing 0.3% Triton X-100 and 2 M NaCl, and the suspension was rotated at 4°C for 5 minutes. After centrifugation at 10,000 rpm for 10 minutes, the supernatant was recovered as fraction 3 (high salt-extractable fraction), and the pellet was boiled with 400 µl Laemmli sample buffer (fraction 4, high salt-resistant fraction).

Western blotting

Western analysis was carried out as previously described[12]. Primary antibodies used in this study were 1:1000-diluted rabbit polyclonal anti-CAP-H2 (Bethyl Laboratories, A302-275A; Abgent, AP1973A), 1:1000-diluted mouse monoclonal anti-p53 (Thermo Fisher Scientific, DO-7), 1:1000-diluted goat polyclonal anti-Lamin B (Santa Cruz Biotechnology, sc-6217), 1:50000-diluted mouse monoclonal anti-α-tubulin (Sigma Aldrich, DM1A), 1:1000-diluted mouse monoclonal anti-Flag (Sigma Aldrich, clone M2), 1:1000-diluted rabbit monoclonal anti-histone H3 (Cell Signaling Technology, #4499), 1:1000-diluted mouse monoclonal anti-IL1B (Santa Cruz Biotechnologies, E7-2-hIL1β), 1:1000-diluted mouse monoclonal anti-p21 (Santa Cruz Biotechnologies, sc-817), 1:1000-diluted mouse monoclonal anti-RB (Cell Signaling Technology, #9309), and 1:1000-diluted mouse monoclonal anti-Cyclin A (Santa Cruz Biotechnology, sc-271682). Secondary antibodies, such as horseradish peroxidase (HRP)-conjugated sheep anti-mouse IgG, HRP-conjugated donkey anti-rabbit IgG (GE Healthcare), and HRP-conjugated rabbit anti-goat IgG (Kirkegaard & Perry Laboratories), were used for detection of target proteins by ECL kits (GE Healthcare).

Supplementary References

1. Aird, K.M. *et al.* HMGB2 orchestrates the chromatin landscape of senescence-associated secretory phenotype gene loci. *J Cell Biol* **215**, 325-334 (2016).
2. Chandra, T. *et al.* Independence of repressive histone marks and chromatin compaction during senescent heterochromatic layer formation. *Mol Cell* **47**, 203-214 (2012).
3. Yang, T. *et al.* HiCRep: assessing the reproducibility of Hi-C data using a stratum-adjusted correlation coefficient. *Genome Res* **27**, 1939-1949 (2017).
4. Chandra, T. *et al.* Global reorganization of the nuclear landscape in senescent cells. *Cell Rep* **10**, 471-483 (2015).
5. Sadaie, M. *et al.* Redistribution of the Lamin B1 genomic binding profile affects rearrangement of heterochromatic domains and SAHF formation during senescence. *Genes Dev* **27**, 1800-1808 (2013).
6. Durand, N.C. *et al.* Juicer Provides a One-Click System for Analyzing Loop-Resolution Hi-C Experiments. *Cell Syst* **3**, 95-98 (2016).
7. Tasdemir, N. *et al.* BRD4 Connects Enhancer Remodeling to Senescence Immune Surveillance. *Cancer Discov* **6**, 612-629 (2016).
8. Bensaude, O. Inhibiting eukaryotic transcription: Which compound to choose? How to evaluate its activity? *Transcription* **2**, 103-108 (2011).
9. Criscione, S.W. *et al.* Reorganization of chromosome architecture in replicative cellular senescence. *Sci Adv* **2**, e1500882 (2016).
10. Jao, C.Y. & Salic, A. Exploring RNA transcription and turnover in vivo by using click chemistry. *Proc Natl Acad Sci U S A* **105**, 15779-15784 (2008).
11. Yildirim, O. Isolation of Nascent Transcripts with Click Chemistry. *Curr Protoc Mol Biol* **111**, 4 24 21-13 (2015).
12. Yokoyama, Y., Zhu, H., Zhang, R. & Noma, K. A novel role for the condensin II complex in cellular senescence. *Cell Cycle* **14**, 2160-2170 (2015).
