# Supplementary material for: Involvement of condensin in cellular senescence through gene regulation and compartmental reorganization
Source: Nat Commun. 2019 Dec 12;10:5688. doi: 10.1038/s41467-019-13604-5 (PMC6908677; doi:10.1038/s41467-019-13604-5)
Supplement: Supplementary file 3 — Reporting Summary [file 41467_2019_13604_MOESM3_ESM.pdf]

nature research

Corresponding author(s): Ken-ichi Noma

Reporting Summary

Nature Research wishes to improve the reproducibility of the work that we publish. This form provides structure for consistency and transparency in reporting. For further information on Nature Research policies, see Authors & Referees and the Editorial Policy Checklist.

Statistical parameters

When statistical analyses are reported, confirm that the following items are present in the relevant location (e.g. figure legend, table legend, main text, or Methods section).

n/a	Confirmed	
☐	☒	The exact sample size (*n*) for each experimental group/condition, given as a discrete number and unit of measurement
☐	☒	An indication of whether measurements were taken from distinct samples or whether the same sample was measured repeatedly
☐	☒	The statistical test(s) used AND whether they are one- or two-sided<br>*Only common tests should be described solely by name; describe more complex techniques in the Methods section.*
☐	☒	A description of all covariates tested
☐	☒	A description of any assumptions or corrections, such as tests of normality and adjustment for multiple comparisons
☐	☒	A full description of the statistics including central tendency (e.g. means) or other basic estimates (e.g. regression coefficient) AND variation (e.g. standard deviation) or associated estimates of uncertainty (e.g. confidence intervals)
☐	☒	For null hypothesis testing, the test statistic (e.g. *F*, *t*, *r*) with confidence intervals, effect sizes, degrees of freedom and *P* value noted<br>*Give P values as exact values whenever suitable.*
☒	☐	For Bayesian analysis, information on the choice of priors and Markov chain Monte Carlo settings
☒	☐	For hierarchical and complex designs, identification of the appropriate level for tests and full reporting of outcomes
☐	☒	Estimates of effect sizes (e.g. Cohen's *d*, Pearson's *r*), indicating how they were calculated
☐	☒	Clearly defined error bars<br>*State explicitly what error bars represent (e.g. SD, SE, CI)*

Our web collection on statistics for biologists may be useful.

Software and code

Policy information about availability of computer code

Data collection	Illumina NextSeq 500 platform was used to obtain 76-bp single-end reads and paired-end reads.
Data analysis	Bowtie2 (v2.2.9) for read alignment; the STAR program (v2.5.2), the RSEM program (v1.2.31) and edgeR for RNA-seq analysis

For manuscripts utilizing custom algorithms or software that are central to the research but not yet described in published literature, software must be made available to editors/reviewers upon request. We strongly encourage code deposition in a community repository (e.g. GitHub). See the Nature Research guidelines for submitting code & software for further information.

Data

Policy information about availability of data

All manuscripts must include a data availability statement. This statement should provide the following information, where applicable:

- Accession codes, unique identifiers, or web links for publicly available datasets
- A list of figures that have associated raw data
- A description of any restrictions on data availability

All data will be made publicly available through the Gene Expression Omnibus (GSE118494).

Field-specific reporting

Please select the best fit for your research. If you are not sure, read the appropriate sections before making your selection.

☒ Life sciences ☐ Behavioural & social sciences ☐ Ecological, evolutionary & environmental sciences

For a reference copy of the document with all sections, see nature.com/authors/policies/ReportingSummary-flat.pdf

Life sciences study design

All studies must disclose on these points even when the disclosure is negative.

Sample size	An in situ Hi-C approach was applied to growing, OIS, RS cells (IMR90 and BJ cells)
Data exclusions	No data were excluded.
Replication	In situ Hi-C experiments were repeated using IMR90 growing, OIS, RS and CAP-H2 KD cells, and BJ growing and RS cells.
Randomization	Not applicable.
Blinding	Not applicable.

Reporting for specific materials, systems and methods

Materials & experimental systems

n/a	Involved in the study
☒	☐ Unique biological materials
☐	☒ Antibodies
☐	☒ Eukaryotic cell lines
☒	☐ Palaeontology
☒	☐ Animals and other organisms
☒	☐ Human research participants

Methods

n/a	Involved in the study
☐	☒ ChIP-seq
☒	☐ Flow cytometry
☒	☐ MRI-based neuroimaging

Antibodies

Antibodies used	Mouse monoclonal anti-FLAG (clone M2, Sigma Aldrich), rabbit polyclonal anti-CAP-H2 (Bethyl Laboratories, A302-275A, A302-276A), rabbit polyclonal anti-SMC1 (Bethyl Laboratories, A300-055A), rabbit polyclonal anti-CTCF (Millipore, 07-729), mouse monoclonal anti-Pol II (Covance, 8WG16), goat polyclonal anti-Lamin B (Santa Cruz Biotechnology, sc-6217), mouse monoclonal anti-alpha-tubulin (Sigma Aldrich, DM1A), rabbit monoclonal anti-histone H3 (Cell Signaling Technology, #4499), mouse monoclonal anti-IL1B (Santa Cruz Biotechnologies, E7-2-hIL1β), mouse monoclonal anti-p21 (Santa Cruz Biotechnologies, sc-817), mouse monoclonal anti-RB (Cell Signaling Technology, #9309), mouse monoclonal anti-Cyclin A (Santa Cruz Biotechnology, sc-271682), and mouse monoclonal anti-TP53 (Thermo Fisher Scientific, DO-7) were used.
Validation	Anti-CAP-H2 (Bethyl Laboratories, A302-275A, A302-276A) and mouse monoclonal anti-TP53 (Thermo Fisher Scientific, DO-7) were validated by RNAi experiments

Eukaryotic cell lines

Policy information about cell lines

Cell line source(s)	IMR90 cells and BJ cells were obtained from ATCC. PD 32 growing and PD 84 senescent IMR90 cells were kindly gifted from the Peter Adams laboratory (Sanford Burnham Prebys Medical Discovery Institute).
Authentication	Karyotyped
Mycoplasma contamination	Not detected
Commonly misidentified lines (See ICLAC register)	N.A.

ChIP-seq

Data deposition

☒ Confirm that both raw and final processed data have been deposited in a public database such as GEO.

☒ Confirm that you have deposited or provided access to graph files (e.g. BED files) for the called peaks.

Data access links<br>*May remain private before publication.*	All data will be made publicly available through the Gene Expression Omnibus (GSE118494).
Files in database submission	ChIPseq_IMR90_CAPH2_Bethyl275_G.fastq.gz<br>ChIPseq_IMR90_CAPH2_Bethyl275_OIS1.fastq.gz<br>ChIPseq_IMR90_CAPH2_Bethyl275_OIS2.fastq.gz<br>ChIPseq_IMR90_CAPH2_FLAG_G.fastq.gz<br>ChIPseq_IMR90_CAPH2_FLAG_OIS1.fastq.gz<br>ChIPseq_IMR90_CAPH2_FLAG_OIS2.fastq.gz<br>ChIPseq_IMR90_CAPH2_Bethyl276_G.fastq.gz<br>ChIPseq_IMR90_CAPH2_Bethyl276_OIS.fastq.gz<br>ChIPseq_IMR90_CTCF_G.fastq.gz<br>ChIPseq_IMR90_CTCF_OIS.fastq.gz<br>ChIPseq_IMR90_SMC1_G.fastq.gz<br>ChIPseq_IMR90_SMC1_OIS.fastq.gz<br>ChIPseq_IMR90_PolII_G.fastq.gz<br>ChIPseq_IMR90_PolII_OIS.fastq.gz<br>ChIPseq_IMR90_IgG_G.fastq.gz<br>ChIPseq_IMR90_IgG_OIS.fastq.gz<br>ChIPseq_IMR90_Input_G.fastq.gz<br>ChIPseq_IMR90_Input_OIS.fastq.gz<br>ChIPseq_IMR90_CAPH2_Triptolide_5min.fastq.gz<br>ChIPseq_IMR90_CAPH2_Triptolide_1hour.fastq.gz<br>ChIPseq_IMR90_CAPH2_alpha-amanitin_30min.fastq.gz<br>ChIPseq_IMR90_CAPH2_alpha-amanitin_1hour.fastq.gz<br>ChIPseq_IMR90_CAPH2_Control_noEU.fastq.gz<br>ChIPseq_IMR90_CAPH2_Bethyl275_G.bw<br>ChIPseq_IMR90_CAPH2_Bethyl275_OIS1.bw<br>ChIPseq_IMR90_CAPH2_Bethyl275_OIS2.bw<br>ChIPseq_IMR90_CAPH2_FLAG_G.bw<br>ChIPseq_IMR90_CAPH2_FLAG_OIS1.bw<br>ChIPseq_IMR90_CAPH2_FLAG_OIS2.bw<br>ChIPseq_IMR90_CAPH2_Bethyl276_G.bw<br>ChIPseq_IMR90_CAPH2_Bethyl276_OIS.bw<br>ChIPseq_IMR90_CTCF_G.bw<br>ChIPseq_IMR90_CTCF_OIS.bw<br>ChIPseq_IMR90_SMC1_G.bw<br>ChIPseq_IMR90_SMC1_OIS.bw<br>ChIPseq_IMR90_PolII_G.bw<br>ChIPseq_IMR90_PolII_OIS.bw<br>ChIPseq_IMR90_IgG_G.bw<br>ChIPseq_IMR90_IgG_OIS.bw<br>ChIPseq_IMR90_Input_G.bw<br>ChIPseq_IMR90_Input_OIS.bw<br>ChIPseq_IMR90_CAPH2_Triptolide_5min.bw<br>ChIPseq_IMR90_CAPH2_Triptolide_1hour.bw<br>ChIPseq_IMR90_CAPH2_alpha-amanitin_30min.bw<br>ChIPseq_IMR90_CAPH2_alpha-amanitin_1hour.bw<br>ChIPseq_IMR90_CAPH2_Control_noEU.bw
Genome browser session<br>(e.g. UCSC)	The link will be provided upon request.

Methodology

Replicates	2 replicas for ChIP-seq with mouse monoclonal anti-FLAG (clone M2, Sigma Aldrich) and rabbit polyclonal anti-CAP-H2 (Bethyl Laboratories, A302-275A)
Sequencing depth	20 million reads per sample
Antibodies	Mouse monoclonal anti-FLAG (clone M2, Sigma Aldrich), rabbit polyclonal anti-CAP-H2 (Bethyl Laboratories, A302-275A, A302-276A), rabbit polyclonal anti-SMC1 (Bethyl Laboratories, A300-055A), rabbit polyclonal anti-CTCF (Millipore, 07-729), mouse monoclonal anti-Pol II (Covance, 8WG16), and mouse monoclonal
Peak calling parameters	ChIP-seq peaks were defined by HOMER software (version 4.8.3) using the option "-center -style factor -F 1 -P 0.0001 -fdr

Peak calling parameters	0.05” for RNA Pol II and TP53, while the alternative option “-center -style histone -F 2 -P 0.0001 -fdr 0.05” was used for other proteins.
Data quality	Described in Supplementary Methods.
Software	Bowtie2 (version 2.2.9), Picard (version 2.7.1) and HOMER software (version 4.8.3) were used.
